# Distinct mechanisms of resistance to fulvestrant treatment dictate level of ER independence and selective response to CDK inhibitors in metastatic breast cancer

**DOI:** 10.1186/s13058-021-01402-1

**Published:** 2021-02-18

**Authors:** Kamila Kaminska, Nina Akrap, Johan Staaf, Carla L. Alves, Anna Ehinger, Anna Ebbesson, Ingrid Hedenfalk, Lukas Beumers, Srinivas Veerla, Katja Harbst, Sidse Ehmsen, Signe Borgquist, Åke Borg, Alejandro Pérez-Fidalgo, Henrik J. Ditzel, Ana Bosch, Gabriella Honeth

**Affiliations:** 1 Division of Oncology, Department of Clinical Sciences Lund, Lund University, Lund, Sweden; 2 Department of Cancer and Inflammation Research, Institute of Molecular Medicine, University of Southern Denmark, Odense, Denmark; 3 Division of Clinical Genetics and Pathology, Department of Laboratory Medicine, Skåne University Hospital, Lund, Sweden; 4 Department of Oncology, Odense University Hospital, Institute of Clinical Research, University of Southern Denmark, Odense, Denmark; 5 Aarhus University Hospital, Aarhus, Denmark; 6 INCLIVA Biomedical Research Institute, Valencia, Spain; 7 Department of Hematology, Oncology and Radiation Physics, Skåne University Hospital, Lund, Sweden

**Keywords:** Metastatic breast cancer, Endocrine treatment resistance, Cell cycle inhibitors, Fulvestrant, Cyclin E2

## Abstract

**Background:**

Resistance to endocrine treatment in metastatic breast cancer is a major clinical challenge. Clinical tools to predict both drug resistance and possible treatment combination approaches to overcome it are lacking. This unmet need is mainly due to the heterogeneity underlying both the mechanisms involved in resistance development and breast cancer itself.

**Methods:**

To study the complexity of the mechanisms involved in the resistance to the selective estrogen receptor degrader (SERD) fulvestrant, we performed comprehensive biomarker analyses using several in vitro models that recapitulate the heterogeneity of developed resistance. We further corroborated our findings in tissue samples from patients treated with fulvestrant.

**Results:**

We found that different in vitro models of fulvestrant resistance show variable stability in their phenotypes, which corresponded with distinct genomic alterations. Notably, the studied models presented adaptation at different cell cycle nodes to facilitate progression through the cell cycle and responded differently to CDK inhibitors. Cyclin E2 overexpression was identified as a biomarker of a persistent fulvestrant-resistant phenotype. Comparison of pre- and post-treatment paired tumor biopsies from patients treated with fulvestrant revealed an upregulation of cyclin E2 upon development of resistance. Moreover, overexpression of this cyclin was found to be a prognostic factor determining resistance to fulvestrant and shorter progression-free survival.

**Conclusions:**

These data highlight the complexity of estrogen receptor positive breast cancer and suggest that the development of diverse resistance mechanisms dictate levels of ER independence and potentially cross-resistance to CDK inhibitors.

**Supplementary Information:**

The online version contains supplementary material available at 10.1186/s13058-021-01402-1.

## Background

Approximately 75% of patients with breast cancer have tumors that are estrogen receptor alpha positive (ER+) and lack amplification of the *ERBB2* gene (HER2−) [1]. In ER+/HER2− (from here on referred to as ER+) metastatic breast cancer patients, endocrine therapy is the mainstay treatment, being its efficacy at least equal to chemotherapy with a better toxicity profile [2]. Therefore, endocrine therapy is the preferred first-line treatment in this context [3].

Nevertheless, all patients with ER+ metastatic breast cancer treated with endocrine therapy will develop endocrine resistance eventually. In fact, ER+ breast cancer may either be unresponsive to endocrine therapy (de novo resistance) or lose endocrine responsiveness over time (acquired endocrine resistance) [4]. From a clinical perspective, de novo and secondary or acquired resistance are terms that have been arbitrarily defined [3]. Given the heterogeneity in mechanisms of endocrine resistance, as well as an increasing number of targeted therapeutics that aim to revert such resistance [5–7], there is a pending need to find feasible and reproducible biomarkers that define endocrine resistance. Several molecular mechanisms have been described as the drivers of resistance to treatment. The loss of ER expression [8], the development of *ESR1* mutations [9], and activation of several signaling pathways such as PI3K or MAPK are among the most relevant resistance mechanisms [10, 11]. This heterogeneity in the resistance development is not only applicable to the progressing disease, but can be also drug-specific, as shown by *ESR1* mutations that confer resistance to aromatase inhibitors but not to selective estrogen receptor degraders (SERDs) [12].

Fulvestrant is a SERD administered in metastatic ER+ breast cancer in both first and subsequent lines. In monotherapy, it has been shown to be at least as efficient as aromatase inhibitors in the first-line metastatic setting [13]. Despite its increasing, albeit delayed, relevance in the ER+ metastatic breast cancer setting, scarce data are available in the literature about fulvestrant-acquired endocrine resistance. Indeed, most of the existing information on endocrine resistance comes either from resistance to aromatase inhibitors [11, 14] or from neoadjuvant trials [15], the latter only depicting de novo mechanisms of resistance.

Here, we report a study of processes that drive acquired resistance to fulvestrant therapy, in which we performed a cross-comparison of various in vitro models. The aim of our study was to understand the complexity of the alterations leading to acquired endocrine resistance focusing on the role of cell cycle alterations and to establish potential therapeutic strategies to overcome it. We show that each model of ER+ breast cancer develops distinct mechanisms of resistance to fulvestrant. This heterogeneity is likely to have an impact on the potential to revert the resistance with different types of therapeutic interventions, such as cell cycle inhibitors. We reveal that cyclin E2 overexpression is a marker for resistance to fulvestrant both in vitro and in patient samples.

## Methods

### Cell lines and drug treatments

Breast cancer cell lines CAMA-1, HCC1428, MCF7, T47D, and ZR-75-1 were obtained from ATCC and EFM-19 from DSMZ. Cells were cultured in DMEM/F12 (CAMA-1, MCF7) or RPMI (EFM-19, HCC1428, T47D, ZR-75-1) medium supplemented with 10% FBS, 1% HEPES, 100 U/ml penicillin, and 100 μg/ml streptomycin. All cell lines were used for a maximum of 25 passages and confirmed as mycoplasma-free using the MycoAlert™ PLUS Mycoplasma Detection Kit (Lonza). Treatments with fulvestrant, 4-hydroxytamoxifen (tamoxifen), palbociclib (all Selleck Chemicals), CDK1/2 inhibitor III (Santa Cruz Biotechnology (SC)), and β-estradiol (Sigma-Aldrich) were conducted as specified below. For estrogen depletion and β-estradiol treatments, cells were cultured in phenol red-free medium using charcoal/dextran treated FBS. All cell culture reagents were obtained from GE Healthcare HyClone™ unless stated otherwise.

### Generation of fulvestrant-resistant and palbociclib-resistant cell line models

Fulvestrant-resistant cell models were generated by chronically exposing breast cancer cell lines to increasing concentrations of fulvestrant from 100 pM to a final concentration of 100 nM. Six fulvestrant-resistant (FR) models were thus generated: CAMA-1-FR, EFM-19-FR, HCC1428-FR, MCF7-FR, T47D-FR, and ZR-75-1-FR, denominated after their respective parental line. For maintenance, fulvestrant-resistant lines were grown in complete medium supplemented with 100 nM fulvestrant. Once generated, fulvestrant-resistant cells were sent for short tandem repeat (STR) profiling (Eurofins Genomics) to confirm cell line origin and purity. STR profiles were matched to the DSMZ database. All fulvestrant-resistant lines matched 100% with their respective original cell line. Fulvestrant-resistant lines were used for a maximum of 15 passages after generation.

Additionally, palbociclib-resistant cell models were generated from CAMA-1 and MCF7 lines by chronically exposing parental and fulvestrant-resistant cells to increasing concentrations of palbociclib from 100 pM to a final concentration of 1 μM. Four palbociclib-resistant (PalbRes) models were generated: CAMA-1-PalbRes, CAMA-1-FR-PalbRes, MCF7-PalbRes, and MCF7-FR-PalbRes. For maintenance, palbociclib-resistant lines were grown in complete medium supplemented with 1 μM palbociclib.

### Proliferation assays

For proliferations assays, cells were plated in 96-well plates 24 h before treatment as specified below. Proliferation was assessed using Sulforhodamine B (SRB) assays. Cells were fixed with 17% (w/v) trichloroacetic acid (Sigma-Aldrich) in PBS for 1 h at 4 °C, washed with water, air-dried and stained with 0.4% (w/v) SRB (Sigma-Aldrich) in 1% acetic acid (Sigma-Aldrich) for 20 min. After washing with 1% acetic acid, SRB dye was dissolved in 10 mM Tris base (Thermo Fisher Scientific) and absorbance was assessed using a FLUOstar Omega reader (BMG Labtech).

xCELLigence system (ACEA, Biosciences) was used to analyze proliferation of T47D and EFM-19 cell lines, allowing real-time monitoring of cell proliferation for extended time. Parental and corresponding fulvestrant-resistant cell lines were seeded in a 96-well E-plate, and measurements were automatically collected for 14 days. Results were expressed as the unitless parameter Cell Index (CI).

To calculate IC50 values, 6-day treatment proliferation data for a range of concentrations (minimum 6-points data curve) for specified drugs was plotted as dose-response curves in GraphPad Prism and analyzed using nonlinear regression three-parameters curve fit model of log(inhibitor) vs. response.

### ER reporter assay

An estrogen-responsive element (ERE) reporter system consisting of three EREs in front of the *Firefly* luciferase gene (3X ERE TATA luc, a gift from Donald McDonnell, Addgene plasmid #11354 [16]) was used to assess ER transcriptional activity. The pRL *Renilla* luciferase control vector with CMV promoter (Promega) was used for normalization. Parental and fulvestrant-resistant cells were plated in 12-well plates, and the next day cells transfected with 600 ng 3X ERE TATA luc and 60 ng pRL *Renilla* using Lipofectamine 2000 (Invitrogen). The day after transfection, one well per condition was left untreated and one well was treated with 100 nM fulvestrant. After 24 h, *Firefly* and *Renilla* luciferase activities were assessed sequentially using the Dual-Luciferase Reporter Assay System (Promega) and a FLUOstar Omega reader.

### RNA extraction, cDNA synthesis, and quantitative RT-PCR

Total RNA was extracted using RNeasy Plus Mini Kit (Qiagen) and quantified using a Nanodrop spectrophotometer (Thermo Fisher Scientific). cDNA was synthesized using the High-Capacity cDNA Reverse Transcription Kit (Thermo Fisher Scientific). Quantitative RT-PCR was performed employing the SsoAdvanced™ Universal SYBR® Green Supermix (Bio-Rad) and analyzed on a CFX96 Touch Real-Time PCR Detection System (Bio-Rad) utilizing in-house qPCR assays. Primers used were as follows: *ACTB* forward primer (fwd) CGTCTTCCCCTCCATCGT, reverse primer (rev) GAAGGTGTGGTGCC; *GREB1* fwd GTGGTAGCCGAGTGGACAAT, rev ATTTGTTTCCAGCCCTCCTT, *IGFBP4* fwd AACTTCCACCCCAAGCAGT, rev GGTCCACACACCAGCACTT, *PGR* fwd GGCATGGTCCTTGGAGGT, rev CCACTGGCTGTGGGAGAG.

### Immunoblots

Total protein was extracted in RIPA buffer (1% Igepal, 0.1% SDS, 0.5% sodium deoxycholate, 0.05 M Tris HCl pH 7.4; all Sigma-Aldrich) supplemented with complete protease inhibitor and phosphatase inhibitor PhosSTOP (both Roche). Protein concentration was quantified using Pierce Coomassie (Bradford) Protein Assay (Thermo Fischer Scientific). TGX stain-free 4–20% gradient gels and Trans­Blot Turbo™ PVDF membranes (both Bio-Rad) were used for western blotting. Specific proteins were detected with the following primary antibodies: β-actin (Sigma-Aldrich, A5316), β-tubulin (Cell Signaling Technologies (CST), 2128), CDK2 (SC, sc-163), CDK4 (CST, 2906), CDK6 (Abcam, ab124821), cyclin D1 (CST, 92G2), cyclin E1 (CST, 4129), cyclin E2 (CST, 4132), ER (Thermo Fisher Scientific, SP1), retinoblastoma protein (Rb) (SC, sc-102), phospho-Rb (Ser807/811) (CST, 8516), and phospho-Rb (Thr821) (Abcam, ab4787) followed by HRP-conjugated anti-rabbit or anti-mouse secondary antibodies (Thermo Fisher Scientific). Signals were detected using Luminata Forte Western HRP Substrate (Merck-Millipore) and a ChemiDoc MP (Bio-Rad). All bands were normalized to total lane protein employing stain-free technology and quantified using the Image Lab™ Software v5.2.1 (Bio-Rad). For clarity, β-actin/β-tubulin loading controls are presented for all western blots.

### Removal of fulvestrant from fulvestrant-resistant lines

Fulvestrant was removed from the media of a subset of fulvestrant-resistant cells. Proliferation assays in response to fulvestrant were set up at weeks one, three, six, and nine. Samples for western blotting were collected at each time-point along with corresponding samples cultured in parallel from parental cells as well as fulvestrant-resistant cells grown in the presence of fulvestrant.

### SNP arrays

Total DNA was extracted from all fulvestrant-resistant models as well as from their respective parental cells using DNeasy Blood & Tissue Kit (Qiagen). Genotyping analysis was performed by AROS Applied Biotechnology A/S using the Illumina GSA array. Data extraction and normalization was performed according to the manufacturer’s instructions for the specific array platform. Log R and B allele frequency data was segmented using ASCAT 2 (PMID: 20837533) to derive allele specific copy numbers. Default parameters were used except: penalty = 70, gamma = 0.65, and SNP6 as platform type for noise reduction. Difference in allele specific copy numbers were computed for each SNP and cell line between the fulvestrant-resistant line and the parental line and displayed in histograms.

### Gene expression array

Total RNA was extracted from parental CAMA-1, MCF7, and T47D cultured either without or with 100 nM fulvestrant for 24 h as well as from CAMA1-FR, MCF7-FR, and T47D-FR cultured with fulvestrant using RNeasy Mini Kit. Global gene expression analysis was performed at the Center for Translational Genomics, Lund University and Clinical Genomics Lund, SciLifeLab using Illumina Whole-Genome HumanHT-12 v4 BeadChip covering 47,231 probes. Each cell line was analyzed in four biological replicates. Data was quantile normalized and filtered for probes with detection *p* value ≤ 0.01 in at least 80% of samples prior to log2 transformation and mean centering across all samples. Illumina probes annotated as poor or without a gene match through a reannotation tool [17] were removed. Normalized gene expression data are displayed in Additional file 1. Correlation pattern analysis was performed in each cell line separately. Expression data (*n* = 12,165 probes) for each cell line was scaled and a pattern vector of up in the parental cell line [1], downregulation in parental cells treated 24 h with fulvestrant (− 1), and upregulation in resistant cells [1] was defined. Pearson correlation was used to calculate correlation coefficients for this pattern vector with all gene expression vectors. A cutoff of 0.7 in addition to a standard deviation cutoff of 0.3 in scaled expression was used to identify significant genes per cell line. Results from MCF7, T47D, and CAMA-1 were merged and genes significant in all three cell lines were identified. To identify common processes enriched in fulvestrant-resistant cells, we subjected the identified gene list to gene ontology annotation using the Database for Annotation, Visualization and Integrated Discovery (DAVID) bioinformatics tool [18, 19], applying standard settings for analysis. Data were visualized using the REVIGO web server [20].

### RNA sequencing

Total RNA was extracted from HCC1428-P cells cultured either without or with 100 nM fulvestrant for 24 h as well as from HCC1428-FR cultured with fulvestrant using RNeasy Mini Kit. RNA sequencing was performed at the Center for Translational Genomics, Lund University and Clinical Genomics Lund, SciLifeLab using a NextSeq 500 (Illumina). Each sample was analyzed in four biological replicates. Data were aligned using HISAT2. Stringtie (PMID: 27560171) was used to generate transcript per million (TPM) counts used in subsequent analyses.

### Mutation analysis

The HCC1428 RNA sequencing data was used to look for mutations in the *ESR1* gene. To create a multi-sample pileup, samtools mpileup was run on all parental (untreated and fulvestrant-treated, total *n* = 8) samples together, against the human reference genome sequence, within the genomic regions corresponding to the exons of the *ESR1* gene. Mutations (SNVs and indels) were then called from the pileup file using VarScan version 2.3.8 command “mpileup2cns” (all options were default with addition of: --min-var-freq 0.1). The procedure was repeated for the resistant samples. The analysis identified four mutations (three SNPs and one indel) present in each of the analyzed samples.

### Cell cycle profiling

MCF7 and CAMA-1 parental and fulvestrant-resistant cells were either untreated or treated with 100 nM fulvestrant for 72 h. Cells were fixed with 70% ethanol, treated with 0.2 mg/ml RNase A and stained with 10 μg/ml propidium iodide (Sigma-Aldrich). Flow cytometry for cell cycle distribution was analyzed on a FACSVerse (BD Biosciences) and further analysis was done using FlowJo v7.6.5.

### Patient materials

For the pre- and post-fulvestrant treatment sample comparison, formalin-fixed, paraffin-embedded (FFPE) metastatic tumor samples from ER+ breast cancer patients treated with fulvestrant were selected by database extraction from the archives of the Department of Oncology at Skåne University Hospital. Only three patients with samples before treatment and after progression to the SERD with sufficient material for staining were found. All clinical samples were coded to maintain patient confidentiality and the use of patient data and material was approved by the Regional Ethics Committee in Lund, Sweden (protocol number 2017/3). Additional file 2 shows patients’ information including ER, PgR, and HER2 expression prior treatment with fulvestrant and upon progression as well as earlier lines of treatment.

For the studies on the impact of cyclin E expression on progression-free (PFS) and overall (OS) survival for patients treated with fulvestrant, FFPE clinical samples from two previously described fulvestrant-treated breast cancer patient cohorts were used [21]. Briefly, patients were selected from the Department of Pathology at Odense University Hospital (OUH). The inclusion criteria for all groups were patients with ER+ breast cancer operated/biopsied at OUH for metastatic disease with complete clinical information as well as pathologic verification that the metastatic lesion was of breast cancer origin. Exclusion criteria were competing cancer(s), cytologic biopsies, or insufficient material in the FFPE block. The metastatic biopsies used for evaluation of cyclins E1 and E2 expression were obtained soon after disease recurrence diagnoses and prior to fulvestrant treatment. The patients belonged to two different cohorts; the first collected between 2009 and 2013 and the second between 2013 and 2016. Survival data was updated for the current analysis. Due to insufficient material from the FFPE block in some cases, the final number of included patients was 35 for cohort 1 and 48 for cohort 2. Because of these small numbers, both cohorts were pooled together for statistical analysis.

### Immunostainings

Paraffin slides were deparaffinized in xylene and rehydrated in graded ethanol. Antigen retrieval was achieved in a pressure cooker (2100 Retriver, Histolab Products AB) using either pH 6 (cyclin E1) or pH 9 (cyclin E2) buffer (Dako Target Retrieval Solution). Stainings were performed using an AutoStainer Plus with Dako FLEX kit and Dako REAL Hematoxylin as counterstain. Primary antibodies, cyclin E1 (ab238081, Abcam) and cyclin E2 (Abcam, ab40890, clone EP454Y), were incubated for 30 min at room temperature. Scoring was performed by an experienced breast pathologist in a blinded manner and calculated as the percentage of cancer cells with nucleus stained at any intensity in a hotspot area containing 200 cancer cells. The cutoff values for high (> 5% for cyclin E1 and > 15% for cyclin E2) versus low (≤ 5% for cyclin E1 and ≤ 15% for cyclin E2) expression were selected based on survival significance in the pooled cohorts.

### Statistical analysis

A two-tailed *t* test or ANOVA were used to calculate statistical differences for in vitro experiments. Survival data from the two patient cohorts previously described [21] were pooled. PFS was defined as the time elapsed from the date of starting fulvestrant treatment to disease progression or death. OS was defined as the time elapsed from the date of starting fulvestrant treatment to the date of death. Patients without progression/death were censored at the date of database retrieval. PFS and OS curves were generated by Kaplan-Meier estimates and differences between groups were evaluated using log-rank test. For multivariate analysis, the Cox proportional hazards regression model was applied to assess the adjusted HR of PFS by expression level of cyclin E2 and relevant clinicopathological features (age at diagnosis of metastasis and site of recurrence) to assess the existence of interactions. For statistical analysis, STATA v14.0 and GraphPad Prism v7.0 was used. *p* values < 0.05 were considered statistically significant.

## Results

### Fulvestrant-resistant breast cancer cells display downregulation of estrogen signaling and proliferate in an estrogen-independent fashion

**Fig. 1:**
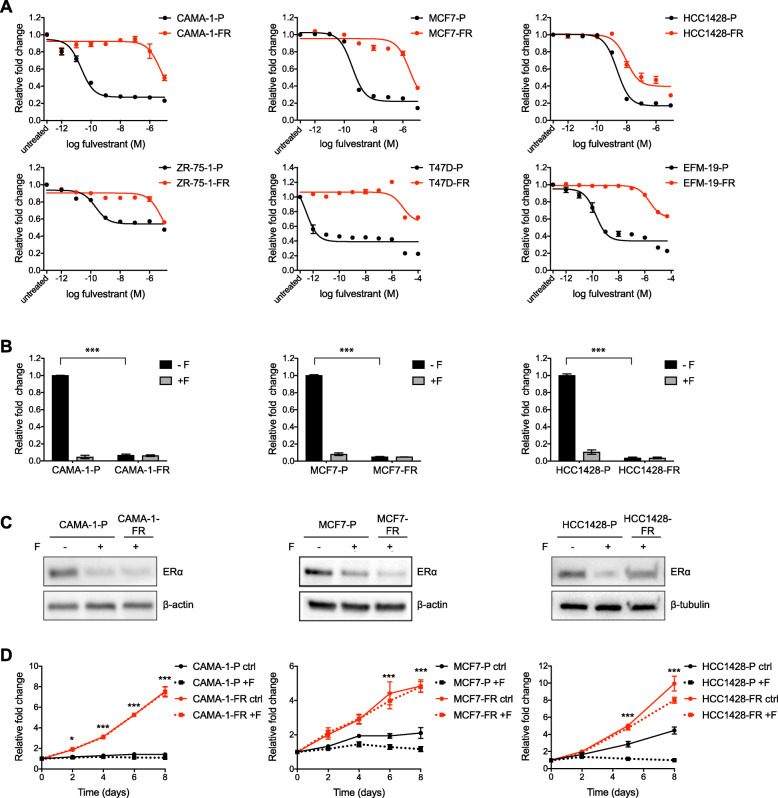
In vitro generated fulvestrant-resistant breast cancer cells downregulate ER signaling and proliferate independently of estrogen. **a** Dose-response curves for parental (-P, black lines) and fulvestrant-resistant (-FR, red lines) cells from six different breast cancer cell lines in response to 6-day treatment with fulvestrant (1 pM to 100 μM). Graphs represent combined data (average ± SEM) from two biological experiments with three technical replicates each. IC50 values are reported in Additional file 3C. **b** ERE reporter activity in parental and fulvestrant-resistant CAMA-1, MCF7, and HCC1428 cells after treatment for 24 h with (+F) or without (-F) supplementation of 100 nM fulvestrant. Graphs represent combined data (average ± SEM) from two biological experiments with two technical replicates each. Statistical differences were determined with one-way ANOVA and Tukey’s post-hoc test, *** represents *p* value ≤ 0.0001 between untreated parental and fulvestrant-resistant cells. Data for the ZR-75-1, T47D, and EFM-19 models are shown in Additional file 3E. **c** Western blotting for ERα expression in parental and fulvestrant-resistant CAMA-1, MCF7, and HCC1428 cells after treatment for 24 h with 100 nM fulvestrant (+F) or DMSO (-F). Representative data from three biological replicates. β-actin or β-tubulin was used as loading control. Quantification of band intensities is presented in Additional file 4A. Data for the ZR-75-1, T47D, and EFM-19 models are shown in Additional file 3F. **d** Proliferation curves for parental and fulvestrant-resistant CAMA-1, MCF7, and HCC1428 cells in estrogen-depleted media with (+F) or without (ctrl) supplementation of 100 nM fulvestrant. Graphs represent combined data (average ± SEM) from two biological experiments with three technical replicates each. Statistical differences were determined with two-way ANOVA and Tukey’s post-hoc test, *** represents *p* value ≤ 0.0001 and * ≤0.05 between untreated parental (black solid lines) and fulvestrant-resistant (red solid lines) cells at given time-points

To establish model systems for studying mechanisms underlying resistance to endocrine therapies, we chronically exposed ER+ breast cancer cell lines to increasing concentrations of fulvestrant during 9 to 40 weeks until the cells overcame their initial sensitivity to this drug and proliferated well in the presence of 100 nM fulvestrant (Additional file 3A-B). Thus, we successfully established fulvestrant-resistant sublines (subsequently denominated with “-FR” after the cell line name) of six breast cancer cell lines: CAMA-1, EFM-19, HCC1428, MCF7, T47D, and ZR-75-1 (parental lines are denominated with “-P” after the cell line name). These models were continuously cultured in the presence of 100 nM fulvestrant. Decreased sensitivity to fulvestrant was confirmed by dose-response proliferation assays (Fig. 1a). IC50 values for fulvestrant increased significantly in all fulvestrant-resistant lines compared to their respective parental line (Additional file 3C).

We characterized the generated fulvestrant-resistant lines in terms of expression patterns for ER and ER target genes. Quantitative RT-PCR analyses demonstrated downregulation of ER target genes *GREB1*, *IGFBP4*, and *PGR* in all fulvestrant-resistant models compared to parental control lines (Additional file 3D). Downregulation of ER signaling was further supported by an ERE reporter gene assay which showed decreased ER activity in all six fulvestrant-resistant models compared to their respective untreated parental line (Fig. 1b and Additional file 3E).

Furthermore, in five out of the six lines, western blotting for ER showed significant downregulation of ER protein expression compared to their respective parental lines (Fig. 1c and Additional file 3F). Surprisingly, the HCC1428-FR model showed persistent high ER protein expression with levels similar to HCC1428-P cells (Fig. 1c). This, however (as shown in Fig. 1b), did not translate into high ER transcriptional activity.

To assess dependence on estrogen for cell proliferation, we evaluated proliferation in three representative fulvestrant-resistant models (CAMA-1-FR, MCF7-FR and HCC1428-FR) as well as their respective parental control lines in estrogen-depleted media (Fig. 1d). Whereas fulvestrant-resistant cells for all these models proliferated well under these conditions, parental cells showed growth arrest.

Thus, our in vitro generated fulvestrant-resistant cell models show phenotypically similar behaviors such as downregulation of ER signaling and estrogen-independent growth.

### Fulvestrant-resistant models display variable stability in their fulvestrant-resistant phenotypes

**Fig. 2:**
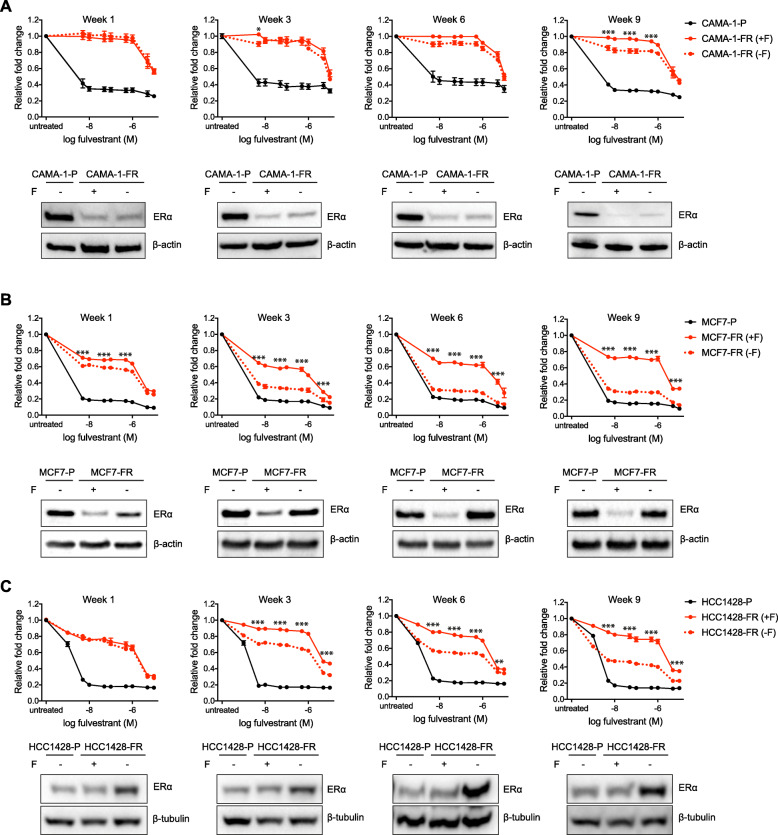
Fulvestrant-resistant models show variable stability upon fulvestrant withdrawal. **a**–**c** Fulvestrant dose-response curves (5 nM to 10 μM) and western blotting for ERα expression in fulvestrant-resistant (-FR) CAMA-1 (**a**), MCF7 (**b**), and HCC1428 (**c**) cells cultured either continuously with fulvestrant (+F, red solid lines) or after removal of fulvestrant (-F, red dotted lines) from the growth media for the indicated times (week 1-week 9). Parental (-P) cells cultured without fulvestrant were used as control (black solid lines). Graphs represent combined data (average ± SEM) from three biological replicates with at least three technical replicates each. Samples for western blotting were collected at each time-point and ERα protein expression was assessed. β-actin or β-tubulin was used as loading control. Representative data from three biological replicates is presented under each graph. Quantification of band intensities is presented in Additional file 4B-D. Stars indicate differences between fulvestrant-resistant cells grown with (red, solid lines) or without (red, dotted lines) fulvestrant. Stars are indicated at every other data point due to restricted space. Data for the ZR-75-1, T47D, and EFM-19 models are shown in Additional file 5

In the clinic, once metastatic breast cancers have developed resistance to endocrine therapy, alone or in combination with a CDK4/6 inhibitor (first and second-line), patients are switched to chemotherapy and the tumors are normally not re-challenged with further endocrine treatments based on the assumption that estrogen signaling has ceased to play a role in cancer survival. To test this assumption, we assessed the stability of the decreased sensitivity to fulvestrant in all our fulvestrant-resistant models by investigating the response to withdrawal of the therapeutic pressure of fulvestrant. These experiments revealed striking differences in response to fulvestrant after up to 9 weeks of culturing the fulvestrant-resistant cells in fulvestrant-depleted conditions (Fig. 2 and Additional file 5). Two of our models (CAMA-1-FR and ZR-75-1-FR) were stable in their fulvestrant-resistant phenotypes (Fig. 2a and Additional file 5A). Up to 6 weeks after removal of fulvestrant from a subpopulation of CAMA-1-FR cells, no differences in response to fulvestrant between cells cultured with or without fulvestrant were observed. Only after 9 weeks without the presence of fulvestrant could we detect a minor increase in response to fulvestrant compared to the CAMA-1-FR cells cultured continuously in the presence of the SERD. For ZR-75-1-FR cells, there was no increase in response to fulvestrant even after being cultured for 9 weeks without fulvestrant. Furthermore, corresponding western blots for ER protein levels showed that ER expression was consistently downregulated in both CAMA-1-FR and ZR-75-1-FR cells for at least 9 weeks after removal of fulvestrant (Fig. 2a and Additional file 5A).

Two of the other models (MCF7-FR and T47D-FR), on the other hand, already reverted their resistance 1 week after removal of fulvestrant and were nearly fully reverted to a similar sensitivity level as their parental lines after 6 weeks of culturing without this drug (Fig. 2b and Additional file 5B). Corresponding western blots for ER protein levels demonstrated that ER expression increased in both these models already 1 week after fulvestrant withdrawal and was comparable to levels in their respective parental lines from 3 weeks (MCF7-FR) or 6 weeks (T47D-FR) and onwards without fulvestrant (Fig. 2b and Additional file 5B).

The remaining two models (HCC1428-FR and EFM-19-FR) did revert to being more fulvestrant sensitive, although they did this more slowly than MCF7-FR and T47D-FR. HCC1428-FR cells started to revert at week 3 without presence of fulvestrant and interestingly kept its higher ER protein levels compared to HCC1428-P throughout the nine weeks of the experiment (Fig. 2c). EFM-19-FR cells started to revert its fulvestrant resistance and re-express ER protein at week 6 without fulvestrant (Additional file 5C).

Thus, our fulvestrant-resistant models could be divided into at least two distinct groups based on their phenotypes after fulvestrant withdrawal, where one group, including the CAMA-1-FR and ZR-75-1-FR models, show a striking stability in their fulvestrant-resistant phenotype while the other four models reverted at different paces and became fulvestrant-sensitive again.

### Fulvestrant-resistant models display different degrees of response to estrogen and tamoxifen stimulation

**Fig. 3:**
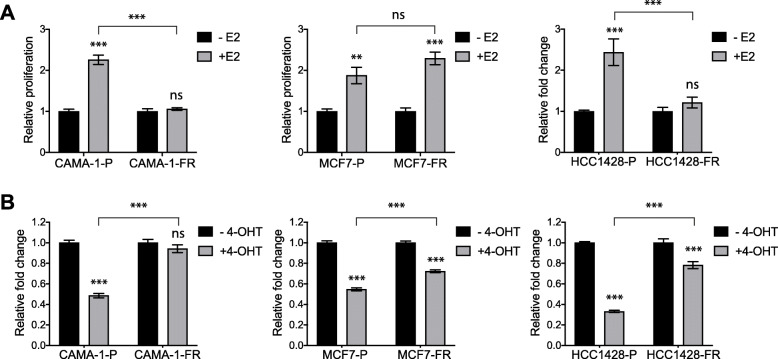
Fulvestrant-resistant models show variable response to estrogen stimulation and tamoxifen treatment. **a**, **b** Relative proliferation of parental and fulvestrant-resistant CAMA-1, MCF7, and HCC1428 cells in estrogen depleted (**a**) or normal (**b**) growth media with or without supplementation with 1 nM estradiol (E2) (**a**) or 100 nM 4-hydroxytamoxifen (4-OHT) (**b**) for 6 days. Each graph represents combined data (average ± SEM) from two biological experiments with three technical replicates each. Statistical differences were determined using one-way ANOVA with Tukey’s post-hoc test. *** represents *p* value ≤ 0.0001, ** ≤ 0.001, ns represents no statistical differences. Stars and “ns” in **a** indicate statistical differences compared to -E2 for each cell model unless indicated otherwise. Stars and “ns” in **b** indicate statistical differences compared to -4-OHT for each cell model unless indicated otherwise. Data for the ZR-75-1, T47D, and EFM-19 models are shown in Additional file 6

To further investigate differences in estrogen signaling, we tested the response to estrogen stimulation in these cell models. Under hormone-depleted conditions, stimulation with 1 nM estradiol (E2) significantly increased cell proliferation in all parental lines as well as in the MCF7-FR model, while all other fulvestrant-resistant models did not respond to E2 stimulation (Fig. 3a and Additional file 6A). Additionally, we assessed the responsivity to the selective estrogen receptor modulator (SERM) tamoxifen. While CAMA-1-FR, EFM-19-FR, T47D-FR, and ZR-75-1-FR cells displayed a significantly decreased sensitivity to this drug compared to their respective parental cells (Fig. 3b and Additional file 6B), surprisingly, MCF7-FR and HCC1428-FR responded to tamoxifen with decreased cell proliferation, although not to the same extent as MCF7-P and HCC1428-P, respectively (Fig. 3b).

Taken together, these data demonstrate that although our models show similar phenotypic properties in terms of development of endocrine resistance and estrogen independent growth, they display a striking heterogeneity not only in terms of stability of endocrine resistance but also in response to estrogen stimulation and tamoxifen treatment, suggesting that they have developed different levels of independence from estrogen.

### Fulvestrant-resistant models present distinct genomic alterations that converge in cell cycle pathway regulation

We performed mutational, genomic, and transcriptional analyses to explore potential molecular reasons behind the heterogeneity displayed by the different fulvestrant-resistant models. Targeted mutation sequencing using a custom bidirectional TRUseq (Illumina) panel of 51 cancer-related genes showed no significant changes between CAMA-1-FR, MCF7-FR, EFM-19-FR, or T47D-FR and their respective parental counterparts. The NGS panel was built on the Illumina TST26 gene content, adding specific exons from 25 genes. Data analysis was performed as previously reported [22]. For the cell lines in question, only variants in *CDH1*, *CDKN2A*, *ERBB4*, *FGFR3*, *GATA3*, *GNAQ*, *KEAP1*, *MAP2K2*, *MAP3K1*, *MEF2A*, *NF1*, *PIK3CA*, *PTEN*, and *TP53* were detected; however, mutational status was not different between resistant and parental cell lines.

**Fig. 4:**
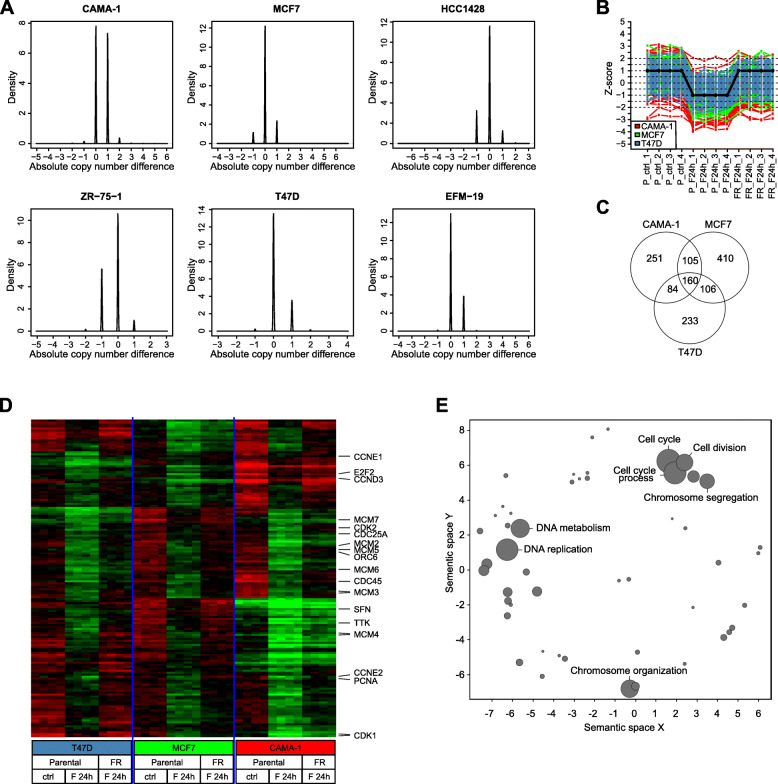
Fulvestrant-resistant models show differences in genomic regulation but similarities in transcriptional output. **a** Histograms of the difference in copy number estimates between fulvestrant-resistant and corresponding parental cell lines for all six models analyzed by Illumina GSA arrays. If no difference exists between cell lines, the histogram would show one narrow spike at 0. Observation of smaller subpopulations of SNP probes with difference in copy number between resistant and parental cells indicates that copy number alterations have occurred. BAF and LogR files are shown in Additional file 7. **b** Graphical illustration of pattern correlation analysis identifying genes that are downregulated in parental cells upon treatment with 100 nM fulvestrant for 24 h, and then upregulated upon development of fulvestrant resistance. **c** Venn diagram of genes following this pattern. One hundred sixty genes were found significant across CAMA-1, MCF7, and T47D models. **d** Gene expression heatmap of 160 genes (rows) found to be significant based on correlation and expression variance cutoffs in the pattern correlation analysis in all three cell lines. Samples (columns) are ordered by (i) cell line, (ii) treatment type (parental control, parental with 24 h fulvestrant treatment (F 24 h), and resistant (FR, F 24 h)), and (iii) biological replicate. Green corresponds to decreased expression (lower limit − 1.5 in expression) and red to increased expression (upper limit 1.5 in expression). Eighteen genes present in the KEGG pathway “Cell cycle” are indicated to the right. All 160 genes are listed in Additional file 1. **e** To determine commonly enriched processes, the list of 160 identified genes was subjected to gene ontology annotation using the DAVID bioinformatics tool (see also Additional file 8). Data were visualized using the REVIGO web server. Data are displayed on the *x*- and *y*-axis based on semantic similarity; hence, more similar GO terms cluster more closely together. The size of each node represents the log10 *p* value of enrichment, the larger the node, the smaller the *p* value, and the more significant the enrichment. Most significantly enriched processes are presented

We also explored copy number alterations (CNA) occurring during development of fulvestrant resistance using single nucleotide polymorphism (SNP) array analysis in all the models. Interestingly, CAMA-1-FR and ZR-75-1-FR showed a larger genomic variation compared to their respective parental lines than the other four models, when comparing actual copy number estimates for all SNP probes across the genome (Fig. 4a). These data suggest that changes in clonal composition may have occurred during development of resistance in CAMA-1-FR and ZR-75-1-FR cells as more copy number alterations have emerged, while MCF7-FR, HCC1428-FR, EFM-19-FR, and T47D-FR may have acquired resistance through adaptation rather than clonal evolution. This could then potentially explain the more stable fulvestrant-resistant phenotypes displayed by the CAMA-1-FR and ZR-75-1-FR models.

Next, we explored whether these differences at the genomic level were also reflected in transcriptional output. For this, we analyzed global gene expression changes occurring in CAMA-1-FR, MCF7-FR and T47D-FR compared with their respective parental cells that were either untreated or treated with 100 nM fulvestrant for 24 h. These analyses revealed distinct regulation of signaling pathways involved in cell cycle regulation, DNA repair, and chromosome functions. When analyzing patterns of genes that were downregulated in parental cells upon treatment with fulvestrant and then upregulated again upon development of resistance, we found 160 genes that followed this pattern in all three cell lines (Fig. 4b-d and Additional file 1). Gene ontology (GO) analysis revealed that the top pathways enriched in this pattern included predominantly pathways involved in cell cycle progression and DNA replication and repair (Fig. 4e and Additional file 8). Genes present in the KEGG pathway “Cell cycle” are indicated in Fig. 4d and include several important cell cycle checkpoint regulators such as *CCND3*, *CCNE1*, *CCNE2*, *CDK1*, and *CDK2*.

Additionally, we investigated transcriptional changes in the HCC1428-FR model using RNA sequencing data from HCC1428-P cells untreated or treated with 100 nM fulvestrant for 24 h and HCC1428-FR cells cultured with fulvestrant. In this model, we found 533 genes that followed the pattern described above of downregulation in fulvestrant-treated parental cells and upregulation upon acquired fulvestrant resistance (Additional file 1), of which 46 genes (including cell cycle regulators *CCND3* and *CCNE2*) were overlapping with the 160 genes described above to follow this pattern in the CAMA-1, MCF7, and T47D models. GO analysis showed that pathways enriched in this pattern in the HCC1428 model were similar to what was seen in CAMA-1, MCF7, and T47D with the top pathways being DNA replication, purine metabolism, and cell cycle regulation (Additional file 8). To investigate if the persisting high ER expression seen in the HCC1428-FR model may be due to an acquired mutation within the ER gene, we furthermore used the RNA sequencing data to look for such mutations, but none could be detected.

These data demonstrate that despite differences in genomic alterations, the fulvestrant-resistant models converge in a common transcriptional phenotype with alterations in cell cycle regulation.

### Fulvestrant-resistant cell models adapt at different cell cycle nodes to facilitate progression through the cell cycle and respond differently to CDK inhibitors

To further assess how our fulvestrant-resistant models progressed through the cell cycle in the absence and presence of fulvestrant, we analyzed cell cycle profiles of CAMA-1-FR and MCF7-FR cells together with parental control. These data showed that while fulvestrant-treated CAMA-1-P and MCF7-P cells arrested in G0/G1 phase, fulvestrant-treated CAMA-1-FR and MCF7-FR cells progressed through the cell cycle similarly to untreated cells (Additional file 9A).

**Fig. 5:**
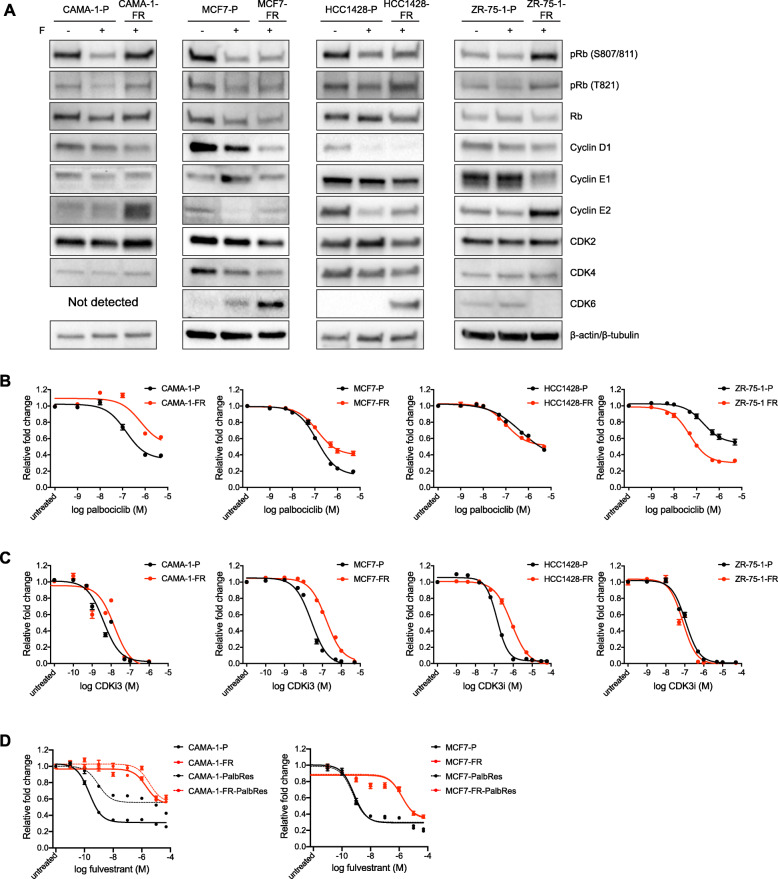
Fulvestrant-resistant models present differential regulation of cell cycle proteins and different sensitivity to CDK inhibition. **a** Western blotting for expression of cell cycle regulating proteins in parental and fulvestrant-resistant CAMA-1, MCF7, HCC1428, and ZR-75-1 cells after treatment for 24 h with 100 nM fulvestrant (+F) or DMSO control (-F). Representative data from three biological replicates. β-actin (CAMA-1, MCF7, and ZR-75-1) or β-tubulin (HCC1428) were used as loading control. Quantification of band intensities is presented in Additional file 4H-K. Data for the T47D and EFM-19 models are shown in Additional file 9B. **b**, **c** Dose-response curves for parental and fulvestrant-resistant CAMA-1, MCF7, HCC1428, and ZR-75-1 cells in response to 6-day treatment with increasing concentrations of the CDK4/6 inhibitor palbociclib (1 nM to 5 μM) (**b**) or a CDK1/2 inhibitor (CDKi3, 100 pM to 50 μM) (**c**). Graphs represent combined data (average ± SEM) from three biological experiments with three technical replicates each. IC50 values are shown in Additional file 9C and E. Data for the T47D and EFM-19 models are shown in Additional file 9D and F. **d** Dose-response curves for parental (-P, black solid lines), fulvestrant-resistant (-FR, red solid lines), palbociclib-resistant (-PalbRes, black dotted lines), and double fulvestrant- and palbociclib-resistant (-FR-PalbRes, red dotted lines) CAMA-1 and MCF7 cells in response to 6-day treatment with fulvestrant (10 pM to 50 μM). Graphs represent combined data (average ± SEM) from minimum two biological experiments with three technical replicates each. IC50 values are shown in Additional file 9J. Dose-response curves in response to palbociclib are shown in Additional file 9H

Western blotting for cell cycle regulators revealed that total levels of the retinoblastoma protein (Rb) were similar in CAMA-1-FR, MCF7-FR, HCC1428-FR, and ZR-75-1-FR cells compared to their parental counterparts (Fig. 5a). The CAMA-1-FR and ZR-75-1-FR models did however display consistently higher Rb phosphorylation levels when compared to the parental counterpart treated with fulvestrant. This difference was not seen in the MCF7-FR and HCC1428-FR models. EFM19-FR and T47D-FR cells, on the other hand, showed downregulation of both total and phosphorylated Rb expression (Additional file 9B).

Furthermore, MCF7-FR and HCC1428-FR cells (Fig. 5a) as well as EFM-19-FR and T47D-FR cells (Additional file 9B) showed upregulated levels of CDK6, a previously described finding in other fulvestrant-resistant models [23]. CDK6 upregulation has been proposed as a driver of resistance to fulvestrant that can be overcome with the use of CDK4/6 inhibitors [21]. In contrast, neither parental nor fulvestrant-resistant CAMA-1 cells exhibited detectable levels of CDK6 while ZR-75-1 expressed very low CDK6 level only in parental cells (Fig. 5a). When treating our different models with the CDK4/6 inhibitor palbociclib, we saw no difference in sensitivity to this compound for MCF7-FR, EFM-19-FR and T47D-FR cells compared to their respective parental cells (numerical differences in IC50 values between parental and FR cells for some of these models are deemed not biologically relevant) (Fig. 5b and Additional file 9C-D). HCC1428-FR and ZR-75-1-FR cells on the other hand were more sensitive to palbociclib than their respective parental cells while CAMA-1-FR cells displayed diminished sensitivity compared to CAMA-1-P cells (Fig. 5b). These data suggest that CDK6 expression levels alone do not determine response to palbociclib in these in vitro models.

Interestingly, both models that did not upregulate CDK6 (i.e., CAMA-1 and ZR-75-1) instead significantly upregulated cyclin E2 upon developing fulvestrant resistance, suggesting that cell cycle progression in these models may be dependent on the cyclin E/CDK2 axis. Furthermore, treatment of the fulvestrant-resistant MCF7, HCC1428, EFM-19, and T47D models with the CDK1/2 inhibitor III (CDKi3) showed less response in terms of growth inhibition compared to their respective parental counterparts, while the CAMA-1-FR and ZR-75-1-FR models (that both upregulated cyclin E2) responded to this drug equally to parental cells, further supporting the importance of the cyclin E/CDK2 axis in these models (numerical differences in IC50 values deemed not biologically relevant) (Fig. 5c and Additional file 9E-F).

To further investigate potential cross-resistance between fulvestrant and palbociclib, we used palbociclib-resistant models derived from MCF7 and CAMA-1 parental and fulvestrant-resistant models. These models were generated through chronic exposure to increasing doses of palbociclib during 11.5 to 22 weeks until cells could proliferate in the presence of 1 μM palbociclib (Additional file 9G). Established palbociclib-resistant sublines (denoted with –PalbRes) displayed decreased sensitivity to palbociclib in comparison to parental cell lines (Additional file 9H-I). We further evaluated response of the palbociclib-resistant sublines to fulvestrant treatment (Fig. 5d and Additional file 9J). As expected, fulvestrant-resistant and double fulvestrant- and palbociclib-resistant models remained poorly responsive to fulvestrant. Furthermore, there was no difference in sensitivity to fulvestrant between MCF7-Palb-Res and MCF7-P cells. In contrast, CAMA-1-PalbRes line displayed reduced response to fulvestrant comparing to CAMA-1-P cells, indicating that there is at least partial cross-resistance between fulvestrant and palbociclib in this model.

Together, these data suggest that the heterogeneity that our in vitro models of fulvestrant resistance show in terms of estrogen growth-dependence may be a result of distinct adaptations at different cell cycle nodes, resulting in diverse response to cell cycle inhibitors.

### Cyclin E2 levels increase after progression on fulvestrant in patient samples and correlate with clinical outcome

**Fig. 6:**
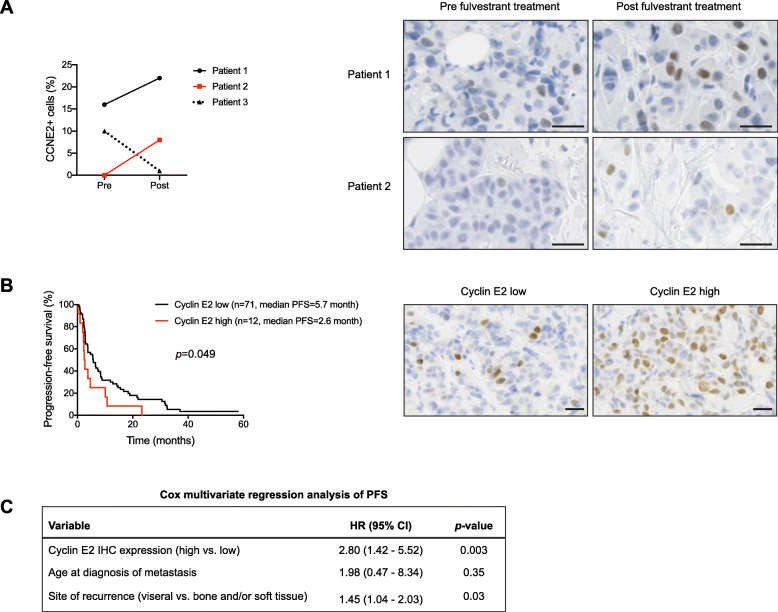
Cyclin E2 levels increase after progression on fulvestrant and correlate with clinical outcome. **a** Representation of cyclin E2 expression in samples from three patients with ER+ breast cancer before initiation of fulvestrant treatment (pre) and upon progress during treatment (post) as determined by immunohistochemical stainings. Quantifications of cyclin E2 scoring for each pre- and post- sample are shown to the left and representative stainings are shown to the right. **b** Evaluation of progression-free survival (PFS) in tumors with cyclin E2 low versus high expression in ER+ breast cancer patients treated with fulvestrant in the advanced setting. Kaplan-Meier plots for PFS are shown to the left and representative stainings for low and high cyclin E2 expression are shown to the right. *p* value represents log-rank test for PFS between patients with high and low levels of cyclin E2 expression. Scale-bar represents 25 μm in (**a**, **b**). **c** Cox multivariate regression analysis of PFS according to cyclin E2 expression levels, age at diagnosis of metastasis, and site of recurrence

To assess the clinical relevance of our findings, we analyzed cyclin E2 expression in tumor samples collected from three patients treated with fulvestrant. Paired tumor biopsies were collected from patients prior to fulvestrant therapy and upon progression to treatment. Notably, two of the patients were diagnosed upfront with advanced breast cancer and did not received chemotherapy prior to the treatment with fulvestrant. Patient 1 was both ER+ and HER2 amplified and, therefore, was treated with trastuzumab concomitantly with fulvestrant. In both these patients, we saw a clear increase in cyclin E2 expression when comparing pre- and post- biopsies (Fig. 6a). The third patient was treated with fulvestrant upfront when diagnosed with a relapse, due to intolerance to aromatase inhibitors in the adjuvant setting. In this patient, cyclin E2 expression did not follow the same trend in the post-progression biopsy. In all three cases, the biopsies done upon fulvestrant failure were still ER+.

To further corroborate the role of cyclin E in resistance to fulvestrant, we analyzed the expression of cyclin E2 by IHC in metastatic tumor samples from a cohort of 83 ER+ breast cancer patients treated with fulvestrant [21]. All samples were collected prior to initiation of fulvestrant treatment. As seen in Fig. 6b, the patient group with tumors that exhibited higher levels of cyclin E2 presented a significantly shorter progression-free survival (PFS) compared to the patient group that exhibited lower levels of cyclin E2, as determined by log-rank test. Cox proportional hazard regression analysis of PFS according to cyclin E2 expression levels and clinicopathologic characteristics, including age at metastatic disease and site of relapse (Fig. 6c), showed that cyclin E2 was an independent prognostic factor for PFS. The difference in PFS did not translate into significant differences in overall survival (OS) (Additional file 10A), probably due to the small patient numbers. Given prior data on the role of *CCNE1* expression as a marker of resistance to palbociclib [24], we additionally analyzed expression of cyclin E1 in the same clinical samples. However, and in concordance to the in vitro data (Fig. 5a), differences in cyclin E1 protein expression did not influence survival in this patient cohort (Additional file 10B-C).

Together, our in vitro and patient data support cyclin E2 overexpression as a biomarker for resistance to fulvestrant.

## Discussion

Resistance development to any given drug is a heterogeneous process. Preclinical studies developed to investigate this complex phenomenon require the construction of various models to ensure a broad perspective and account for different molecular processes that result in disease progression. By using several in vitro models of resistance to fulvestrant, we demonstrate that, although all resistant cell lines presented with some comparable traits such as downregulation of ER signaling and estrogen independent growth, different cell line models showed distinct cross-resistance patterns to other types of ER targeting drugs as well as varying degrees of responsiveness to estradiol. Most of our models showed cross-resistance to tamoxifen and no response to estradiol exposure, while fulvestrant-resistant MCF7 and HCC1428 cells did still respond to tamoxifen and fulvestrant-resistant MCF7 cells also responded to estradiol stimulation. The fulvestrant-resistant HCC1428 model interestingly maintained a high ER expression, regardless of showing a clear resistance to fulvestrant. ER downstream signaling and activity was however downregulated, suggesting that ER is not active in this model, at least not while remaining under fulvestrant pressure. This is in sharp contrast with other models of resistance to endocrine treatment, namely long-term estrogen-deprived (LTED) models, which mimic resistance to aromatase inhibitors and display upregulation of ER and hypersensitivity to estradiol [25]. It should be noted that this model is less sensitive to fulvestrant from the beginning compared to the other used models. It is however still much more sensitive than all the developed resistant models.

Given these differences, we sought to explore the existence of genomic and transcriptomic alterations behind drug resistance. We did not detect mutational differences based on targeted sequencing of 51 cancer-related genes in any of our models, which included *ESR1*. This further corroborates that resistance to endocrine treatment is not only tumor dependent but drug dependent, since it has now been established that *ESR1* mutations are a frequent mechanism of resistance to aromatase inhibition [26, 27]. On the other hand, we observed a large number of copy number differences between resistant and parental lines in the CAMA-1 and ZR-75-1 models based on SNP array analysis. In this context, SNP arrays have been used to identify clonal evolution based on either copy number alone or by integration of also allele specific copy number status [28, 29]. Our observation could indicate that a change has occurred in the clonal disposition in these two models during resistance development. The other four models on the other hand showed fewer copy number changes, indicating that these models may have taken another route in developing resistance. An intriguing observation in this context is that all these latter models reverted to an ER dependent/fulvestrant-sensitive model when cultured without fulvestrant while the CAMA-1 and ZR-75-1 models did not, which could indicate presence of a more “hard-wired” resistance phenotype in the two latter models. Taken together, these data demonstrate that treatment resistance is a very heterogeneous process also in the in vitro setting, mimicking what is seen in patient materials, and suggest that resistance could possibly be a result of either clonal evolution or cellular adaptation. These changes may have an impact on both dependence on ER for cell growth and the subsequent therapeutic approaches to be taken to achieve disease control once fulvestrant resistance develops.

Interestingly, regardless of the genomic evolution the cells underwent, the resistance patterns shared a similar transcriptional phenotype with all the studied models displaying changes in cell cycle regulation in order to become resistant to fulvestrant. Based on previous evidences of cell cycle regulation as a strategy to enhance endocrine treatment activity [5, 6], we aimed to study whether there was a common cell cycle regulator that could be targeted in order to reverse resistance. In this context, and as already described [23], we confirmed that four of our six models upregulated CDK6 expression upon developing fulvestrant resistance. CDK6 has been postulated as a driver of resistance to fulvestrant but also a biomarker of response to CDK4/6 inhibitors that when used in fulvestrant resistant models, will help revert the resistant phenotype [21]. Furthermore, CDK4/6 inhibitors have been identified also in the clinic as a valuable alternative for overcoming endocrine treatment resistance [30–33], although some conflicting clinical data exists [34]. Clearly, there is a need to address resistance to endocrine treatment as a heterogeneous process and consider that the current clinical definition of resistance falls short when it comes to choosing consecutive treatment for these patients.

In this context, all our models that upregulated CDK6 also remained sensitive to the CDK4/6 inhibitor palbociclib. Fulvestrant-resistant CAMA-1 and ZR-75-1 cells, on the other hand, did not upregulate CDK6. Both these models instead show an upregulation of cyclin E2 and sustained response to a CDK1/2 inhibitor while all the other fulvestrant-resistant models responded less to this drug than their respective parental counterpart. These data indicate that fulvestrant-resistant CAMA-1 and ZR-75-1 cells may have developed dependency on the CDK2/cyclin E axis for cell growth. Interestingly, these two latter models were also the ones that showed a more stable fulvestrant-resistant phenotype with numerous genomic alterations. This suggests cyclin E2 upregulation as a marker for a persistent fulvestrant-resistant phenotype in vitro.

Further from this, we saw the same pattern of cyclin E2 upregulation upon development of resistance to fulvestrant in two out of three metastatic breast cancer patients with paired biopsies before and after fulvestrant treatment. The main limitation from these data is that the pre-treatment biopsy was not taken directly before fulvestrant treatment; therefore, we cannot conclude with certainty that upregulation of cyclin E2 was a direct consequence of progression to the SERD. However, in a larger cohort of patients, we also found that higher expression of cyclin E2 correlated with shorter PFS in response to fulvestrant. While this is still a small number of patients, the data support that cyclin E2 expression is increased when the tumor develops resistance to fulvestrant and should be further studied as a biomarker of resistance to fulvestrant in the clinical setting.

Upregulation of cyclin E has been postulated as marker of resistance to CDK4/6 inhibitors [24]; however, these results have not been confirmed in other patient cohorts. Our gene expression data found high levels of *CCNE1* and *CCNE2* in our fulvestrant-resistant models, but we could only detect upregulation at the protein level for cyclin E2 in two of our models. The presented data from these two models suggests that overexpression of this cyclin is a biomarker of irreversible resistance to SERDs and true estrogen independence. This in turn may have an impact on efficacy of CDK4/6 inhibitors. Acquired resistance to SERDs may translate into decreased sensitivity to CDK4/6 inhibitors in a subgroup of ER+ tumors where cancer cells have developed a complete independence from ER for growth and survival. In the clinical setting, these findings could have an important impact as cyclin E2 expression might potentially identify those patients that will not benefit from treatment with estrogen receptor degraders and where the addition of CDK4/6 inhibitors will not reverse this resistance to endocrine treatment.

## Conclusions

Our data demonstrate that endocrine resistance to fulvestrant is a heterogeneous process mediated by either ER independence and/or modification of cell cycle regulators. In this context, the association of CDK4/6 inhibitors to fulvestrant or other antiestrogens needs to be carefully assessed, since, depending on the mechanisms of resistance to endocrine therapies, these cell cycle inhibitors might or might not be useful for reverting resistance. Our findings highlight the importance of disease follow-up to understand the dynamics of acquired resistance to SERDs and to make appropriate therapeutic approaches when endocrine resistance emerges.

## Supplementary Information

**Additional file 1.** Table showing normalized gene expression data from parental and fulvestrant-resistant CAMA-1, MCF7, T47D and HCC1428 cell lines.

**Additional file 2.** Table showing patient characteristics for patient samples pre and post fulvestrant treatment.

**Additional file 3. **Figure showing that fulvestrant-resistant cells proliferate in the presence of fulvestrant and downregulate ER signaling. A) Time in weeks for each parental cell line to develop resistance to fulvestrant, from initial 100 pM dose until able to proliferate in presence of 100 nM fulvestrant. B) Proliferation curves for parental (-P, black lines) and fulvestrant-resistant (-FR, red lines) cells in the absence (ctrl, solid lines) or presence (+F, dotted lines) of 100 nM fulvestrant assessed using SRB assays (CAMA-1, MCF7, HCC1428, ZR-75-1) or xCELLigence system (T47D, EFM-19). Graphs represent combined data (average ± SEM) from two (xCELLigence) or three (SRB) biological experiments with at least three technical replicates each. Statistical differences were determined with two-way ANOVA and Tukey’s post-hoc test. * represents *p*-value ≤0.01, ** ≤0.001 and *** ≤0.0001 between fulvestrant-treated parental (black dotted lines) and fulvestrant-resistant (red dotted lines) cells. C) Fulvestrant IC50 values in parental and fulvestrant-resistant cells. Calculated from graphs presented in Fig. 1a. *P*-values were calculated using Extra sum-of-squares *F* test. D) Quantitative RT-PCR analysis of RNA expression for the ER downstream target genes insulin like growth factor binding protein 4 (*IGFBP4*), growth regulation by estrogen in breast cancer 1 (*GREB1*) and progesterone receptor (*PGR*) in parental and fulvestrant-resistant cells after 24-h treatment with 100 nM fulvestrant (+F) or no treatment (ctrl). Bar graphs represent average expression (± SEM) from two biological experiments with three technical replicates each, normalized against *ACTB* expression and set relative to untreated parental cells. Statistical differences were determined with one-way ANOVA and Dunnett’s post-hoc test, *** represents *p*-value ≤0.001 compared to respective untreated parental control. E) ERE reporter activity in parental and fulvestrant-resistant ZR-75-1, T47D and EFM-19 cells after treatment for 24 h with (+F) or without (-F) supplementation of 100 nM fulvestrant. Graphs represent combined data (average ± SEM) from two biological experiments with two technical replicates each. Statistical differences were determined with one-way ANOVA and Tukey’s post-hoc test, *** represents *p*-value ≤0.0001 between untreated parental and fulvestrant-resistant cells. F) Western blotting for ERα expression in parental and fulvestrant-resistant ZR-75-1, T47D and EFM-19 cells after treatment for 24 h with 100 nM fulvestrant (+F) or DMSO (-F). Representative data from three biological replicates. β-actin was used as loading control. Quantification of band intensities is presented in Additional file 4A.

**Additional file 4. **Figure showing quantification of western blotting band intensities. Quantification of western blotting band intensities presented in Fig. 1c, 2a-c and 5a as well as Additional Files 3F, 5A-C and 9B. Combined data from at least three biological replicates. Bands were normalized to total lane protein and set relative to untreated parental cells, except for CDK6 in (J, L and M) that where set relative to fulvestrant-resistant cells. Statistical differences were determined with one-way ANOVA and Dunnett’s post-hoc test, * represents *p*-value ≤0.05, ** ≤0.01 and *** ≤0.001 compared to respective untreated parental control.

**Additional file 5.** Figure showing fulvestrant-withdrawal data for the ZR-75-1, T47D and EFM-19 models. Fulvestrant dose-response curves (5 nM to 10 μM) and western blotting for ERα expression in fulvestrant-resistant (-FR) ZR-75-1 (A), T47D (B) and EFM-19 (C) cells cultured either continuously with fulvestrant (+F, red solid lines) or after removal of fulvestrant (-F, red dotted lines) from the growth media for the indicated times (Week 1-Week 9). Parental (-P) cells cultured without fulvestrant were used as control (black solid lines). Graphs represent combined data (average ± SEM) from three biological replicates with at least three technical replicates each. Samples for western blotting were collected at each time-point and ERα protein expression was assessed. β-actin was used as loading control. Representative data from three biological replicates is presented under each graph. Quantification of band intensities is presented in Additional file 4E-G. Stars indicate differences between fulvestrant-resistant cells grown with (red, solid lines) or without (red, dotted lines) fulvestrant. Stars are indicated at every other data point due to restricted space.

**Additional file 6. **Figure showing estradiol and tamoxifen response in the ZR-75-1, T47D and EFM-19 models. Relative proliferation of parental and fulvestrant-resistant cells in estrogen depleted (A) or normal (B) growth media with or without supplementation with 1 nM estradiol (E2) (A) or 100 nM 4-hydroxytamoxifen (4-OHT) (B) for 6 days. Each graph represents combined data (average ± SEM) from two biological experiments with three technical replicates each. Statistical differences were determined using one-way ANOVA with Tukey’s post-hoc test. *** represents *p*-value ≤0.0001, ** ≤0.001, ns represents no statistical differences. Stars and ‘ns’ in (A) indicate statistical differences compared to -E2 for each cell model unless indicated otherwise. Stars and ‘ns’ in (B) indicate statistical differences compared to -4-OHT for each cell model unless indicated otherwise.

**Additional file 7.** Figure showing BAF and LogR files for copy number data. B allele frequency (BAF) and LogR genome-wide plots for SNP profiled cell line data presented in Fig. 4a. Chromosomes are ordered along the x-axis from 1 (left) to Y (right). Green lines represent segments derived from ASCAT 2 segmentation of the data.

**Additional file 8.** Table showing common processes enriched in fulvestrant-resistant cells identified by DAVID bioinformatics tool. DAVID functional categories and gene ontology (GO) functions as well as KEGG and Biocarta pathways for genes downregulated upon fulvestrant treatment in parental cells and then upregulated again upon development of resistance. The first table show data for CAMA-1, MCF7 and T47D and the second table show data HCC1428.

**Additional file 9. **Figure showing cell cycle regulation and response to CDK inhibitors. A) Cell cycle profiles for parental (-P) and fulvestrant-resistant (-FR) CAMA-1 and MCF7 cells showing the proportion of cells in G0/G1, S, and G2/M phase, respectively, after treatment with 100 nM fulvestrant for 72 h (+F) or untreated (ctrl). Combined data from two biological replicates. Statistical differences between the proportion of cells in G0/G1-phase were calculated using student’s *t*-test. * represents *p*-value ≤0.05, ns represents no statistical differences. B) Western blotting for expression of cell cycle regulating proteins in parental and fulvestrant-resistant T47D and EFM-19 cells after treatment for 24 h with 100 nM fulvestrant (+F) or DMSO control (-F). Representative data from three biological replicates. β-actin was used as loading control. Quantification of band intensities is presented in Additional file 4 L-M. C) Palbociclib IC50 values in parental and fulvestrant-resistant cells. Calculated from graphs presented in (D) and in Fig. 5b. D) Dose-response curves for parental and fulvestrant-resistant T47D and EFM-19 cells in response to 6-days treatment with increasing concentrations of the CDK4/6 inhibitor palbociclib (1 nM to 10 μM). Graphs represent combined data (average ± SEM) from three biological experiments with three technical replicates each. IC50 values are shown in (C). E) CDKi3 IC50 values in parental and fulvestrant-resistant cells. Calculated from graphs presented in (F) and in Fig. 5c. F) Dose-response curves for parental and fulvestrant-resistant T47D and EFM-19 cells in response to 6-days treatment with increasing concentrations of the CDK1/2 inhibitor CDKi3 (100 pM to 50 μM). Graphs represent combined data (average ± SEM) from three biological experiments with three technical replicates each. IC50 values are shown in (E). G) Time in weeks needed for each parental and fulvestrant-resistant cell line to develop resistance to palbociclib, counted from initial exposure to 100 pM dose until the cells were able to proliferate in presence of 1 μM palbociclib. H) Dose-response curves for parental (-P, solid black lines), fulvestrant-resistant (-FR, solid red lines), palbociclib-resistant (-PalbRes, black dotted lines) and double fulvestrant- and palbociclib-resistant (-FR-PalbRes, red dotted lines) CAMA-1 and MCF7 cells in response to 6-days treatment with palbociclib (1 pM to 10 μM). Graphs represent combined data (average ± SEM) from two biological experiments with three technical replicates each. IC50 values are shown (I). I) Palbociclib IC50 values for graphs in (H). J) Fulvestrant IC50 values for graphs presented in Fig. 5d. *P*-values in (C, E, I and J) were calculated using Extra sum-of-squares *F* test.

**Additional file 10. **Figure showing survival analyses of cyclin E1 and E2 in fulvestrant-treated metastatic breast cancer patients. Kaplan-Meier plot for overall survival (OS) in cyclin E2 low versus high expression in ER+ metastatic breast cancer patients treated with fulvestrant in the advanced setting (A) and for progression-free (PFS) (B) and overall (C) survival in cyclin E1 low versus high expression in the same patient material. *P*-value represents log-rank test for OS and PFS, respectively, between patients with high and low levels of cyclin E1 or E2 expression.

## Data Availability

All data generated or analyzed during this study are included in the published article and its supplementary information. The data for gene expression and RNA sequencing are included in Additional file 1.

## Abbreviations

CCN: Cyclin
CDK: Cyclin-dependent kinase
CNA: Copy number alterations
E2: Estradiol
ER: Estrogen receptor
ERBB2: Erb-B2 receptor tyrosine kinase 2
ESR1: Estrogen receptor 1
FBS: Fetal bovine serum
FFPE: Formalin-fixed paraffin-embedded
FR: Fulvestrant-resistant
GO: Gene ontology
HER2: Human epidermal growth factor receptor 2
IHC: Immunohistochemistry
MAPK: Mitogen-activated protein kinase
OS: Overall survival
P: Parental
PFS: Progression-free survival
PI3K: Phosphoinositide 3-kinase
Rb: Retinoblastoma protein
SERD: Selective estrogen receptor degrader
SERM: Selective estrogen receptor modulator
SNP: Single nucleotide polymorphism
SRB: Sulforhodamine B
STR: Short tandem repeat
