# Supplementary material for: Distinct mechanisms of resistance to fulvestrant treatment dictate level of ER independence and selective response to CDK inhibitors in metastatic breast cancer
Source: Breast Cancer Res. 2021 Feb 18;23:26. doi: 10.1186/s13058-021-01402-1 (PMC7893923; doi:10.1186/s13058-021-01402-1)
Supplement: Supplementary file 2 — Additional file 2. Table showing patient characteristics for patient samples pre and post fulvestrant treatment. [file 13058_2021_1402_MOESM2_ESM.pdf]

Patient characteristics for patient samples pre and post fulvestrant treatment.

	PATIENT 1		PATIENT 2		PATIENT 3	
	**pre**	**post**	**pre**	**post**	**pre**	**post**
**Biopsy site**	Breast primary tumour	Skin locoregional	Breast inoperable disease	Breast	Breast recurrence	Skin
**ER status***	40%	15%	100%	90%	100%	90%
**PgR status***	40%	0%	70%	0%	80%	90%
**HER2 status****	3+amplified	3+ amplified	2+ non-amplified	2+ non-amplified	1+	0/1+
**Ki67 status***	36%	50%	24%	40%	38%	13%
**Treatments prior to fulvestrant**	Letrozole, tamoxifen, exemestane		Letrozole		None	

* Percentage positive tumour cells for each marker
** Scored according to ASCO/CAP guidelines
