# Supplementary material for: Distinct mechanisms of resistance to fulvestrant treatment dictate level of ER independence and selective response to CDK inhibitors in metastatic breast cancer
Source: Breast Cancer Res. 2021 Feb 18;23:26. doi: 10.1186/s13058-021-01402-1 (PMC7893923; doi:10.1186/s13058-021-01402-1)
Supplement: Supplementary file 3 — Additional file 3. Figure showing that fulvestrant-resistant cells proliferate in the presence of fulvestrant and downregulate ER signaling. A) Time in weeks for each parental cell line to develop resistance to fulvestrant, from initial 100 pM dose until able to proliferate in presence of 100 nM fulvestrant. B) Proliferation curves for parental (-P, black lines) and fulvestrant-resistant (-FR, red lines) cells in the absence (ctrl, solid lines) or presence (+F, dotted lines) of 100 nM fulvestrant assessed using SRB assays (CAMA-1, MCF7, HCC1428, ZR-75-1) or xCELLigence system (T47D, EFM-19). Graphs represent combined data (average ± SEM) from two (xCELLigence) or three (SRB) biological experiments with at least three technical replicates each. Statistical differences were determined with two-way ANOVA and Tukey’s post-hoc test. * represents p-value ≤0.01, ** ≤0.001 and *** ≤0.0001 between fulvestrant-treated parental (black dotted lines) and fulvestrant-resistant (red dotted lines) cells. C) Fulvestrant IC50 values in parental and fulvestrant-resistant cells. Calculated from graphs presented in Fig. 1a. P-values were calculated using Extra sum-of-squares F test. D) Quantitative RT-PCR analysis of RNA expression for the ER downstream target genes insulin like growth factor binding protein 4 (IGFBP4), growth regulation by estrogen in breast cancer 1 (GREB1) and progesterone receptor (PGR) in parental and fulvestrant-resistant cells after 24-h treatment with 100 nM fulvestrant (+F) or no treatment (ctrl). Bar graphs represent average expression (± SEM) from two biological experiments with three technical replicates each, normalized against ACTB expression and set relative to untreated parental cells. Statistical differences were determined with one-way ANOVA and Dunnett’s post-hoc test, *** represents p-value ≤0.001 compared to respective untreated parental control. E) ERE reporter activity in parental and fulvestrant-resistant ZR-75-1, T47D and EFM-19 cells after trea [file 13058_2021_1402_MOESM3_ESM.pdf]

A

TIME TO DEVELOPE RESISTANCE

	Weeks to resistance
CAMA-1	11
MCF7	28
HCC1428	25
ZR-75-1	9
T47D	40
EFM-19	14

B

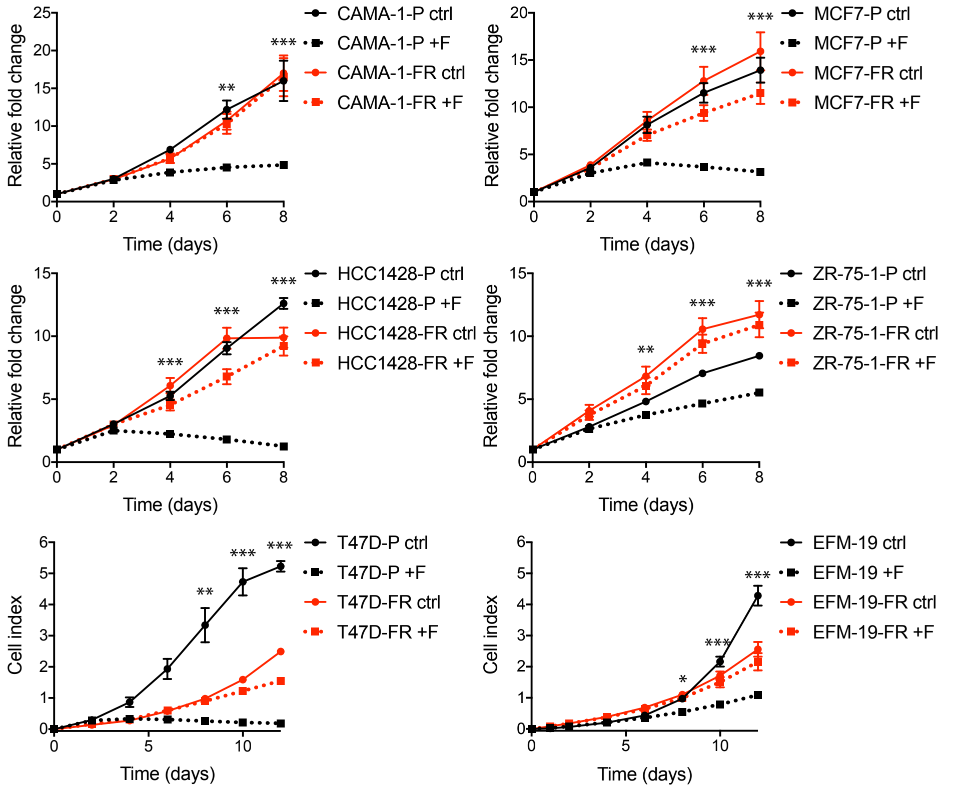

C

IC50 VALUES FULVESTRANT

	Parental	FR	*p*-value
CAMA-1	21 pM	4.9 µM	<0.0001
MCF7	351 pM	2.9 µM	<0.0001
HCC1428	2.2 nM	9.6 nM	<0.0001
ZR-75-1	212 pM	6.2 µM	<0.0001
T47D	0.3 pM	6.7 µM	<0.0001
EFM-19	183 pM	2.3 µM	<0.0001

D

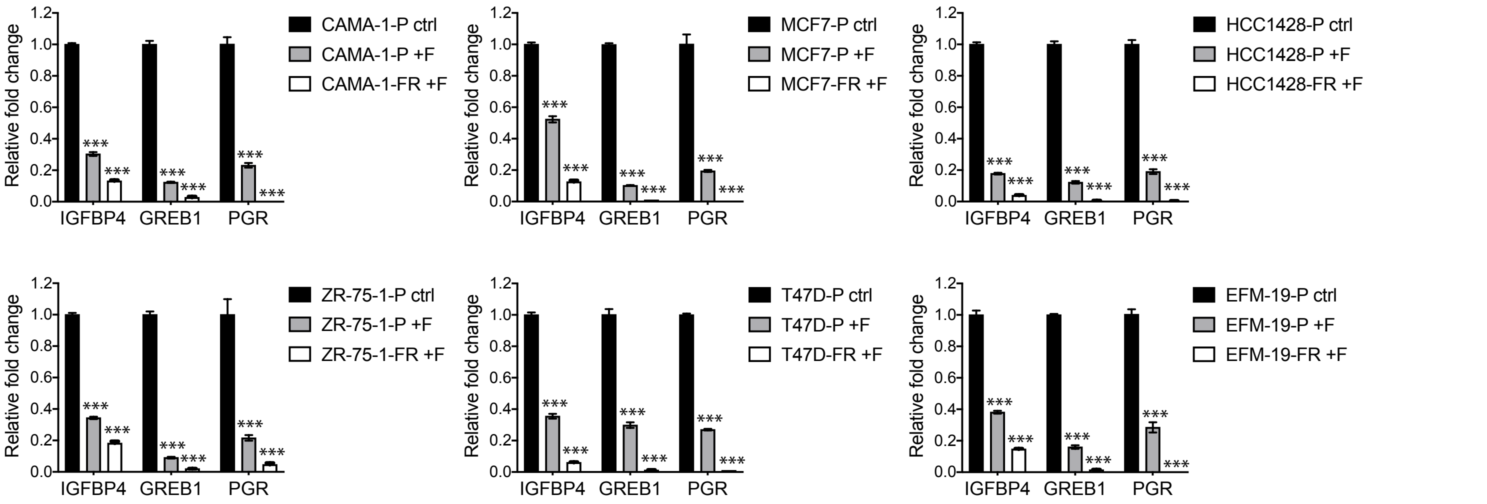

E

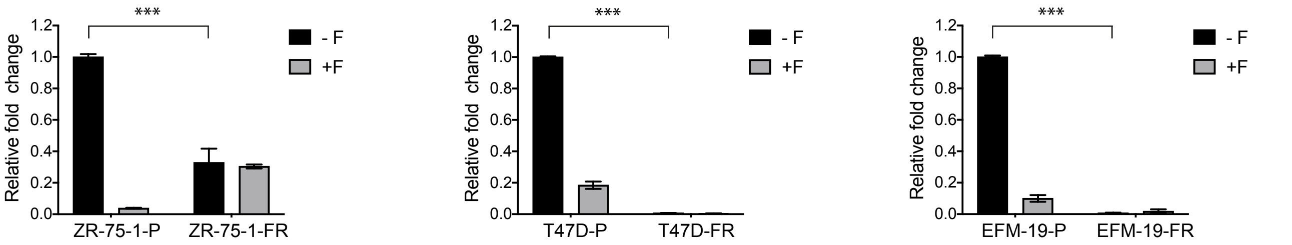

F

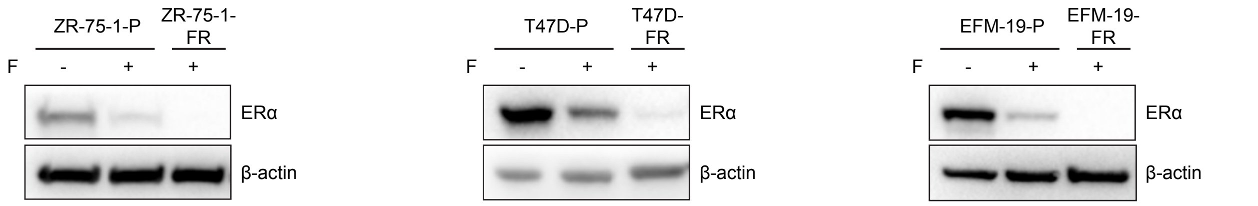

Additional file 3
