# Supplementary figures and images for: Distinct mechanisms of resistance to fulvestrant treatment dictate level of ER independence and selective response to CDK inhibitors in metastatic breast cancer
Source: Breast Cancer Res. 2021 Feb 18;23:26. doi: 10.1186/s13058-021-01402-1 (PMC7893923; doi:10.1186/s13058-021-01402-1)

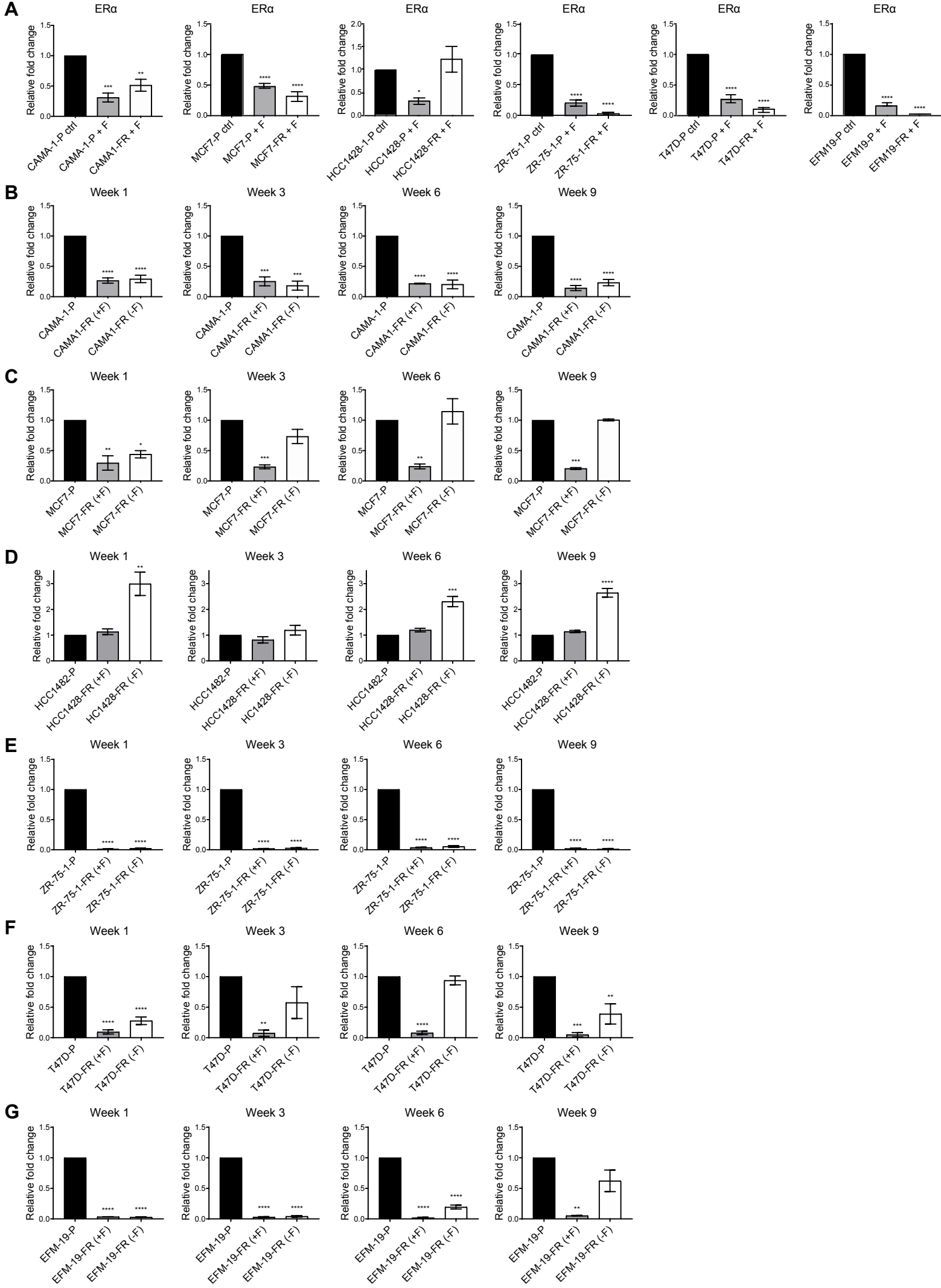

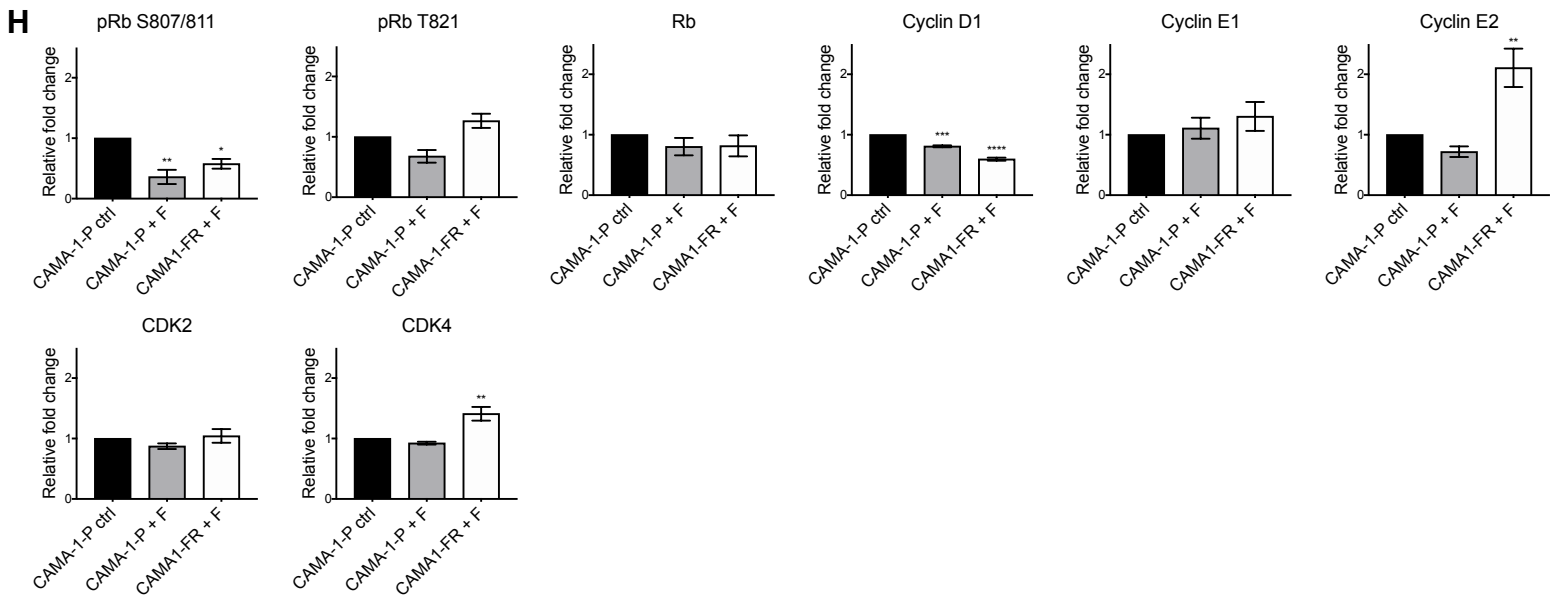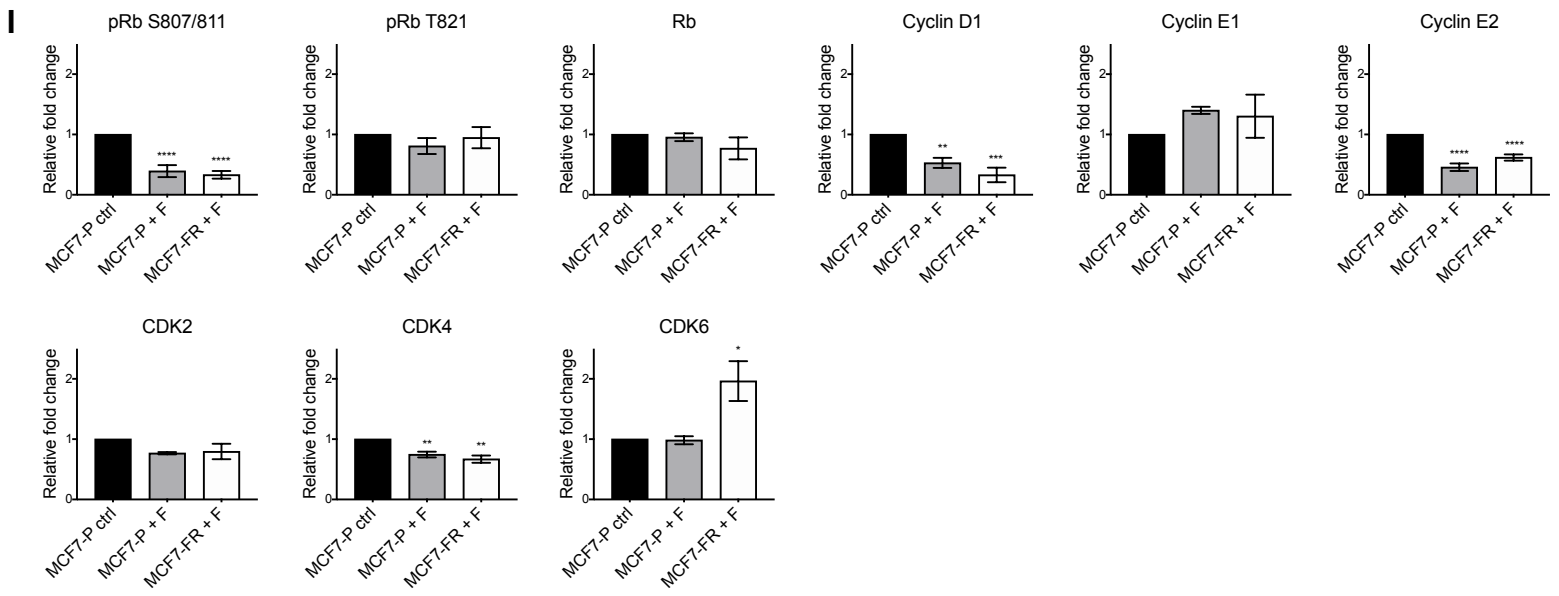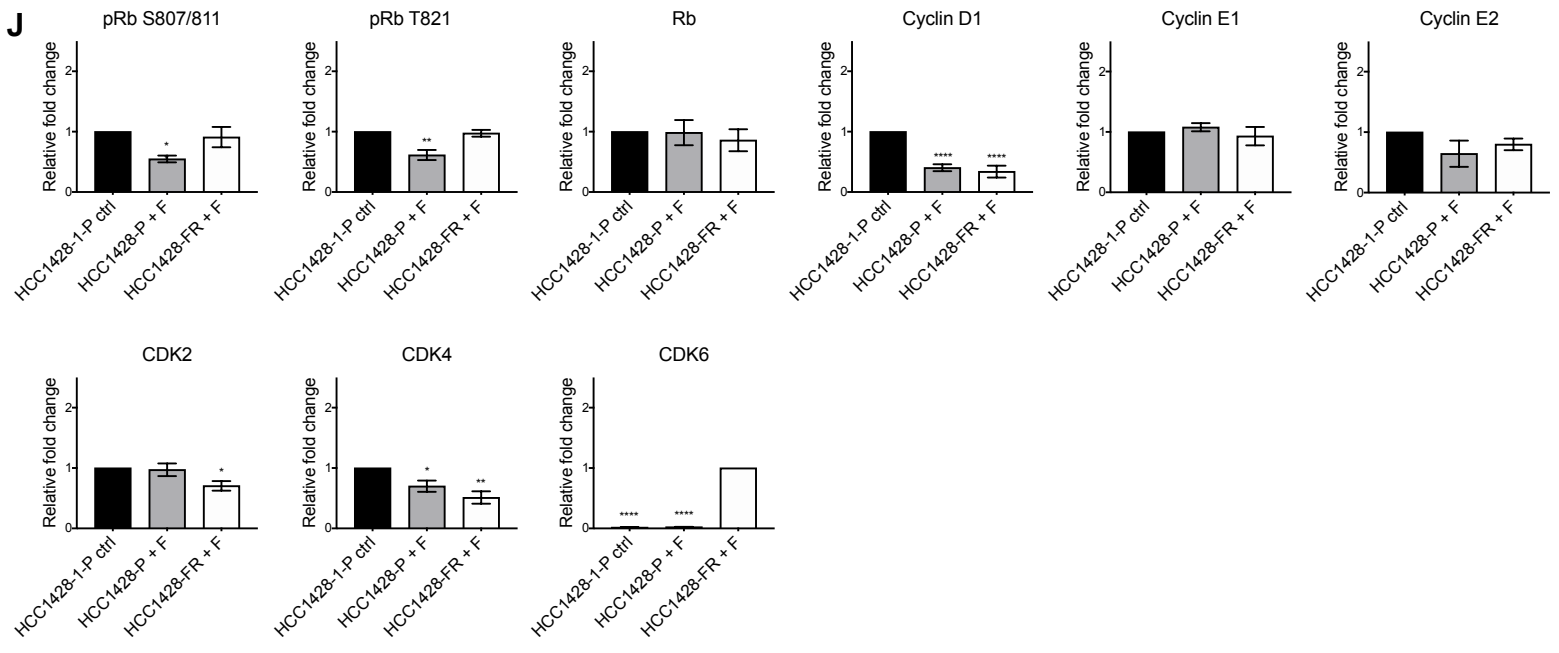

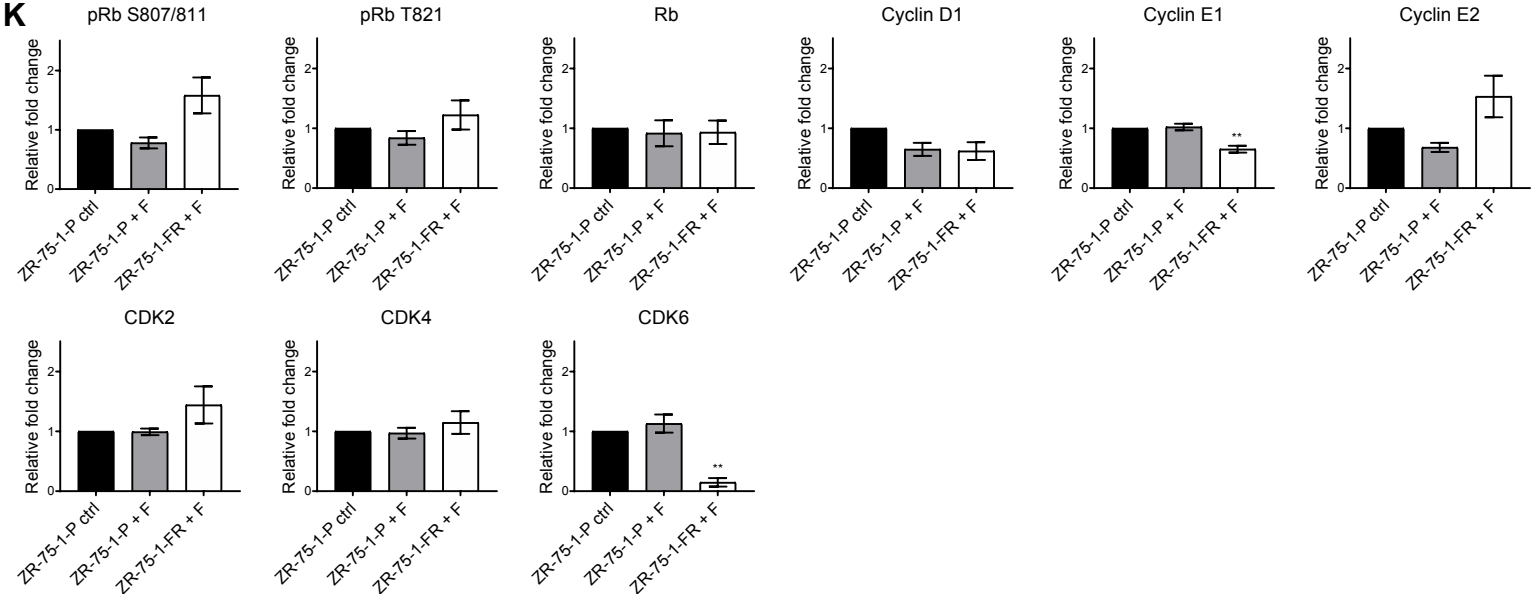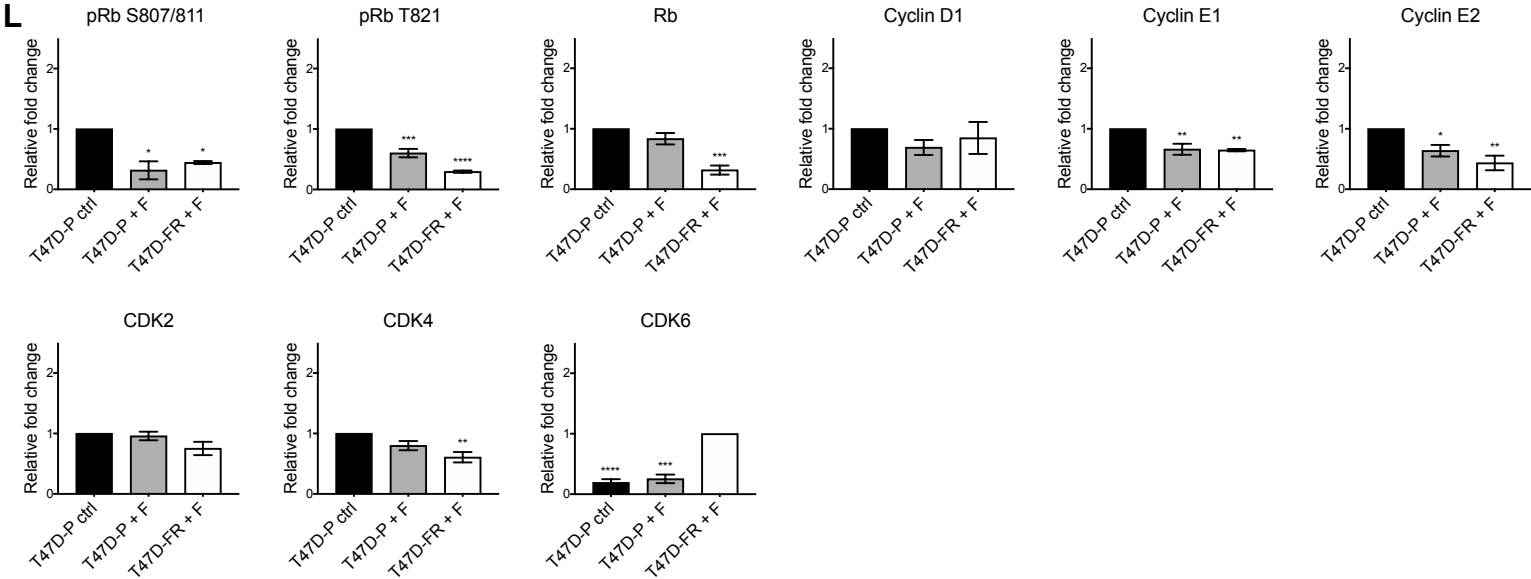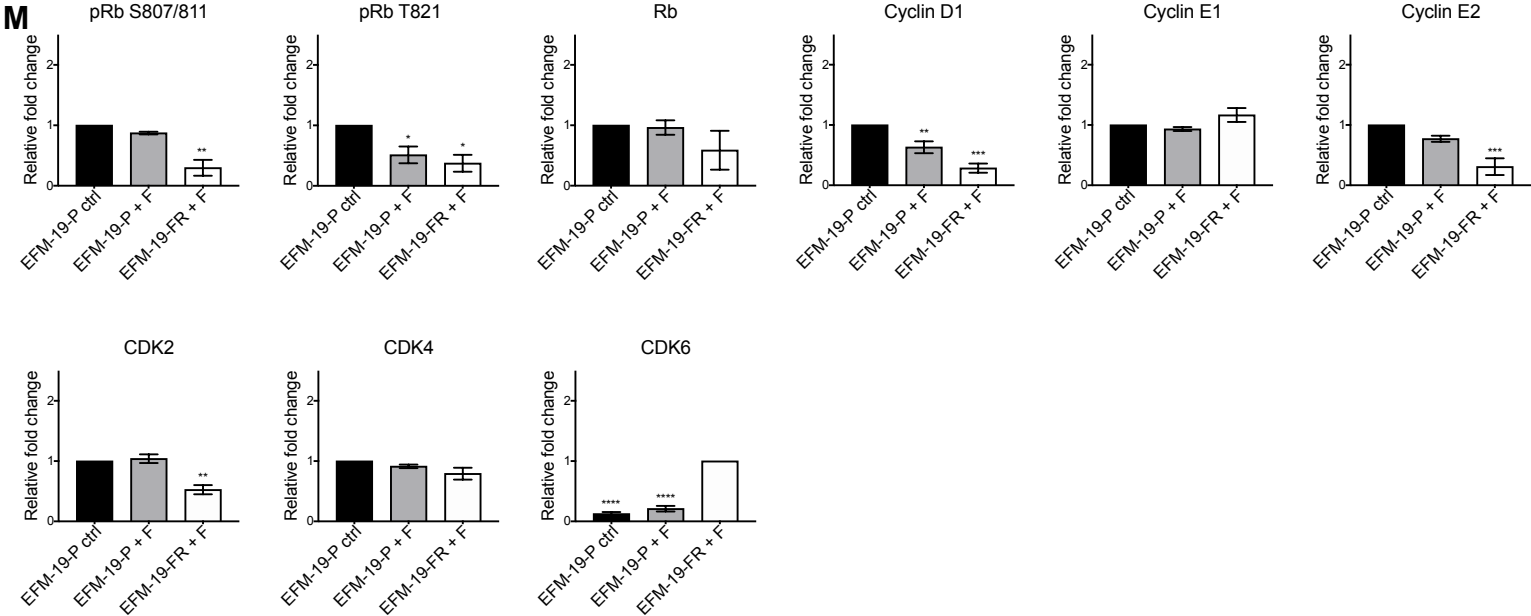

Supplement: Supplementary file 4 — Additional file 4. Figure showing quantification of western blotting band intensities. Quantification of western blotting band intensities presented in Fig. 1c, 2a-c and 5a as well as Additional Files 3F, 5A-C and 9B. Combined data from at least three biological replicates. Bands were normalized to total lane protein and set relative to untreated parental cells, except for CDK6 in (J, L and M) that where set relative to fulvestrant-resistant cells. Statistical differences were determined with one-way ANOVA and Dunnett’s post-hoc test, * represents p-value ≤0.05, ** ≤0.01 and *** ≤0.001 compared to respective untreated parental control. [file 13058_2021_1402_MOESM4_ESM.pdf]

**A**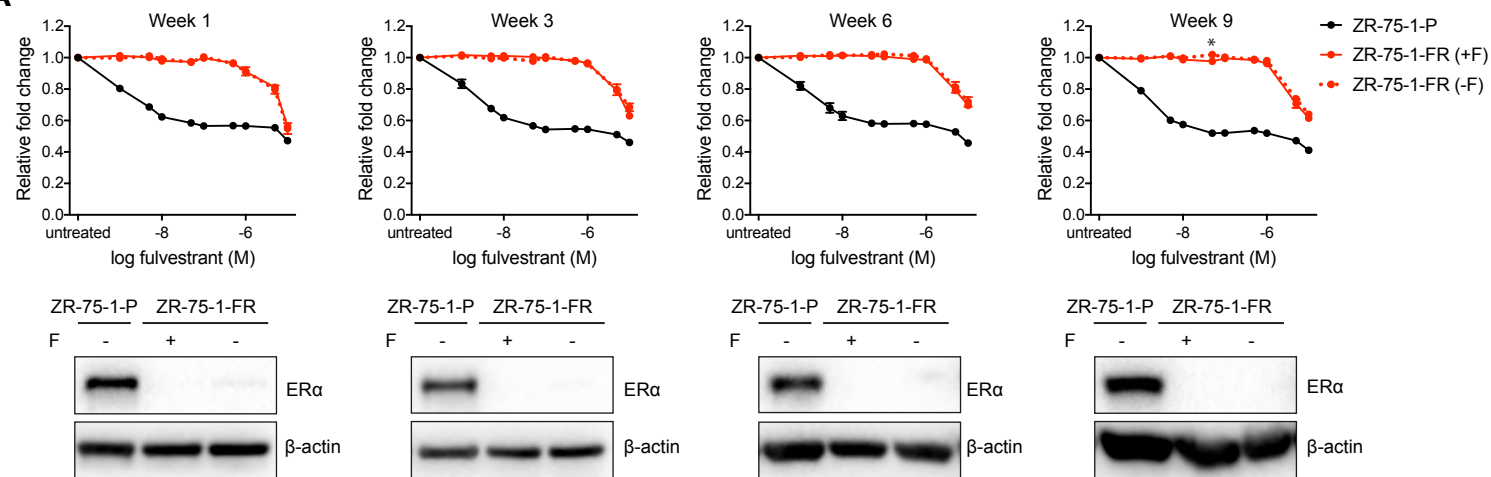**B**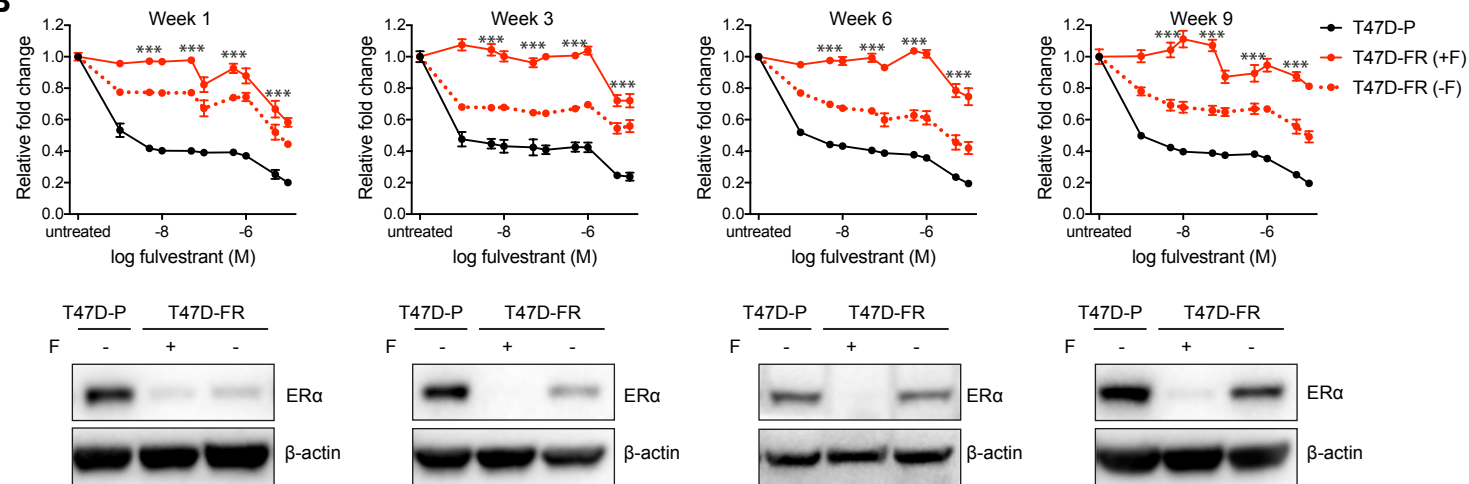**C**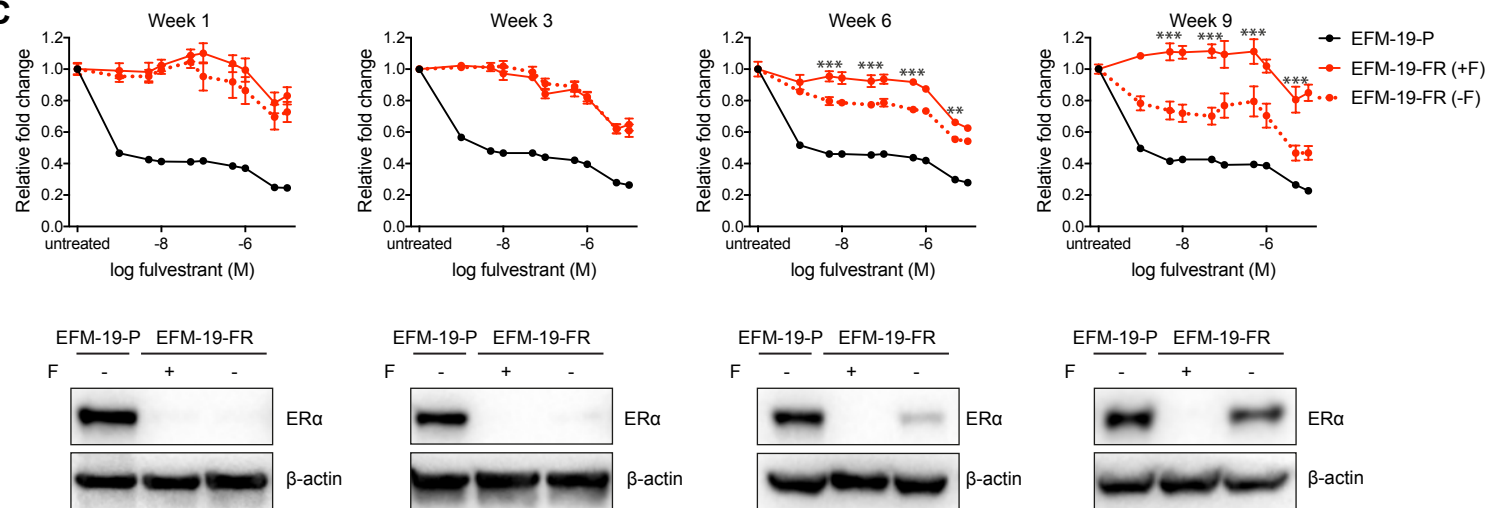

Supplement: Supplementary file 5 — Additional file 5. Figure showing fulvestrant-withdrawal data for the ZR-75-1, T47D and EFM-19 models. Fulvestrant dose-response curves (5 nM to 10 μM) and western blotting for ERα expression in fulvestrant-resistant (-FR) ZR-75-1 (A), T47D (B) and EFM-19 (C) cells cultured either continuously with fulvestrant (+F, red solid lines) or after removal of fulvestrant (-F, red dotted lines) from the growth media for the indicated times (Week 1-Week 9). Parental (-P) cells cultured without fulvestrant were used as control (black solid lines). Graphs represent combined data (average ± SEM) from three biological replicates with at least three technical replicates each. Samples for western blotting were collected at each time-point and ERα protein expression was assessed. β-actin was used as loading control. Representative data from three biological replicates is presented under each graph. Quantification of band intensities is presented in Additional file 4E-G. Stars indicate differences between fulvestrant-resistant cells grown with (red, solid lines) or without (red, dotted lines) fulvestrant. Stars are indicated at every other data point due to restricted space. [file 13058_2021_1402_MOESM5_ESM.pdf]

**A**

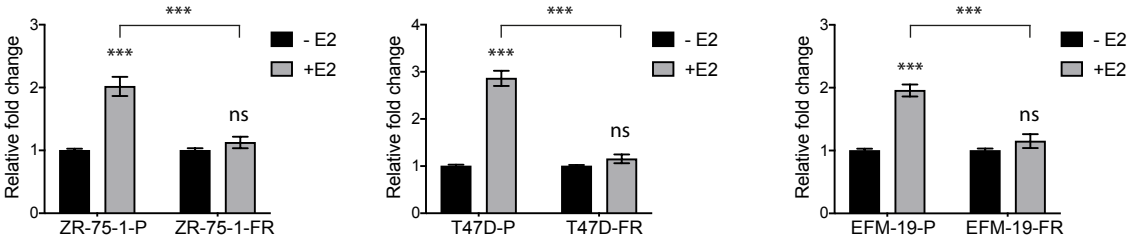

**B**

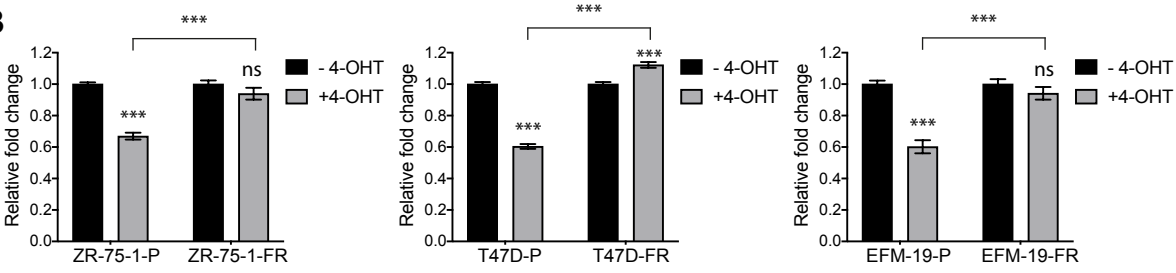

Supplement: Supplementary file 6 — Additional file 6. Figure showing estradiol and tamoxifen response in the ZR-75-1, T47D and EFM-19 models. Relative proliferation of parental and fulvestrant-resistant cells in estrogen depleted (A) or normal (B) growth media with or without supplementation with 1 nM estradiol (E2) (A) or 100 nM 4-hydroxytamoxifen (4-OHT) (B) for 6 days. Each graph represents combined data (average ± SEM) from two biological experiments with three technical replicates each. Statistical differences were determined using one-way ANOVA with Tukey’s post-hoc test. *** represents p-value ≤0.0001, ** ≤0.001, ns represents no statistical differences. Stars and ‘ns’ in (A) indicate statistical differences compared to -E2 for each cell model unless indicated otherwise. Stars and ‘ns’ in (B) indicate statistical differences compared to -4-OHT for each cell model unless indicated otherwise. [file 13058_2021_1402_MOESM6_ESM.pdf]

**A**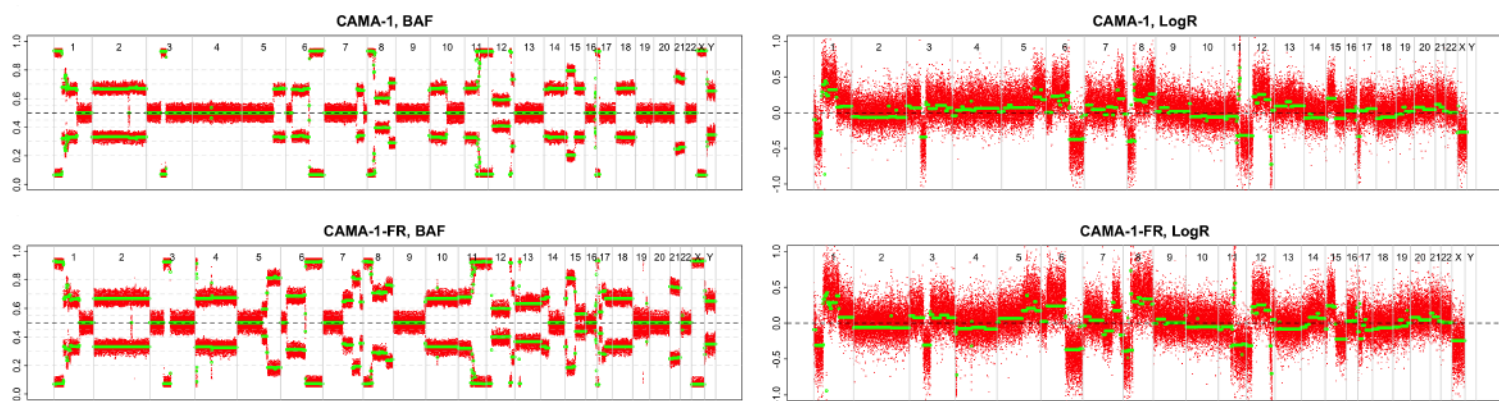**B**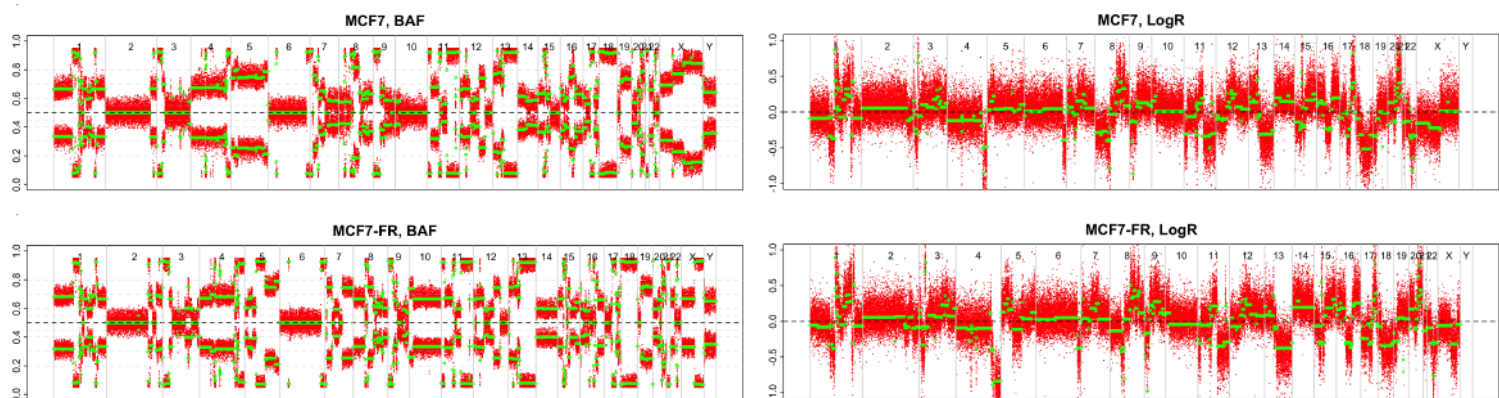**C**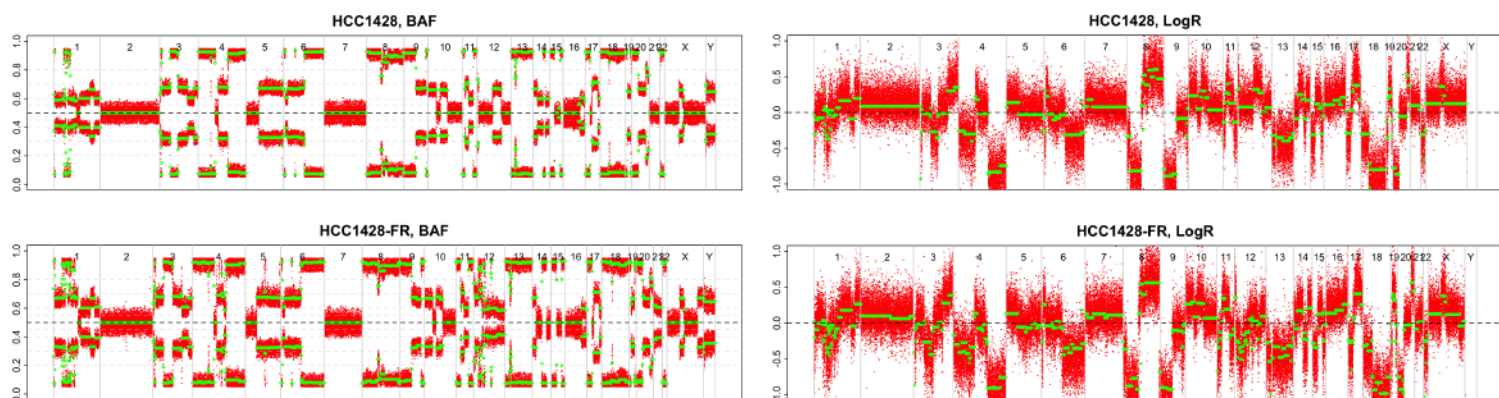**D**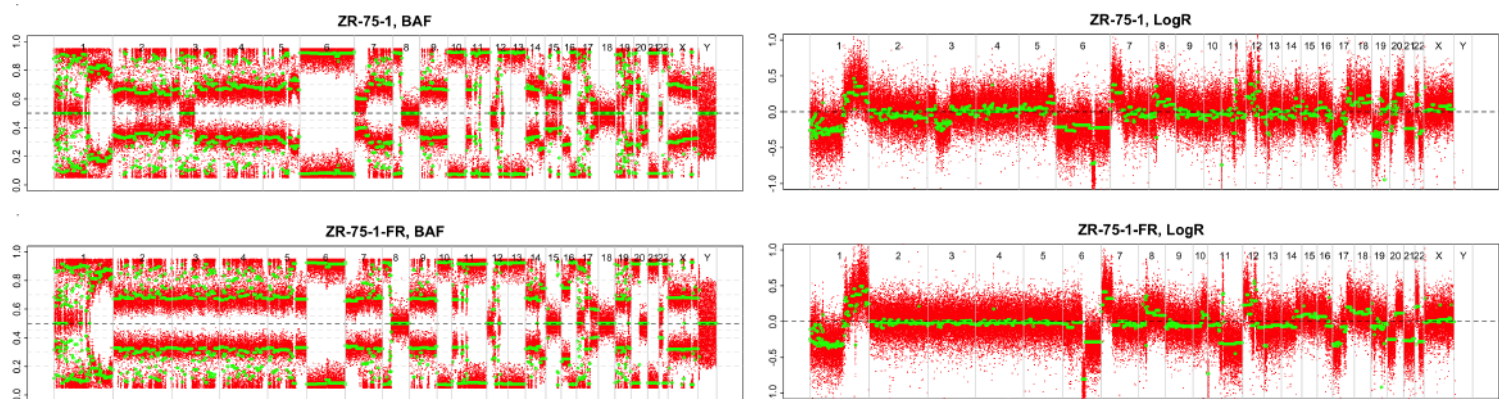

**E**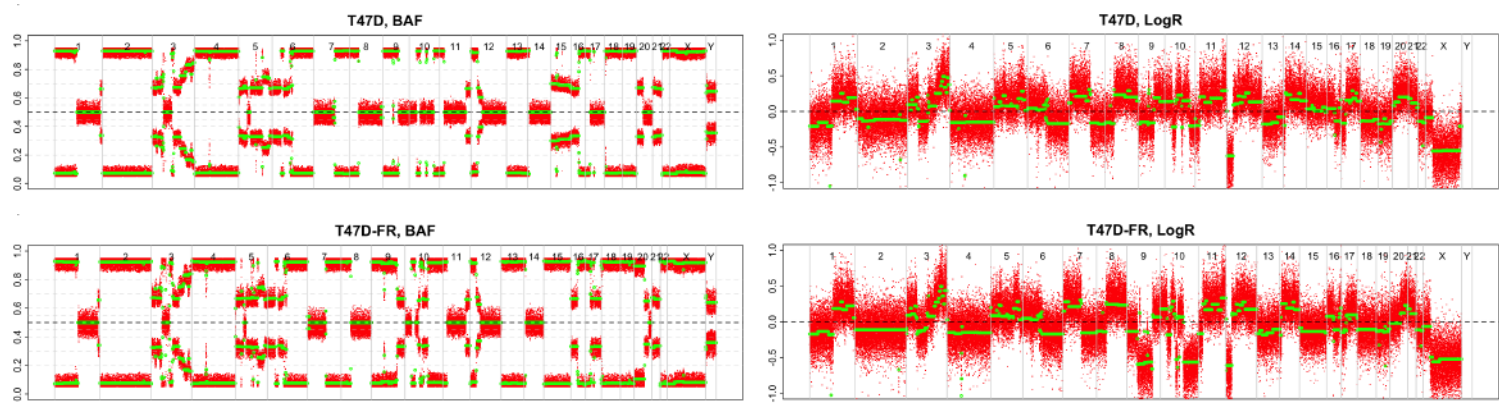**F**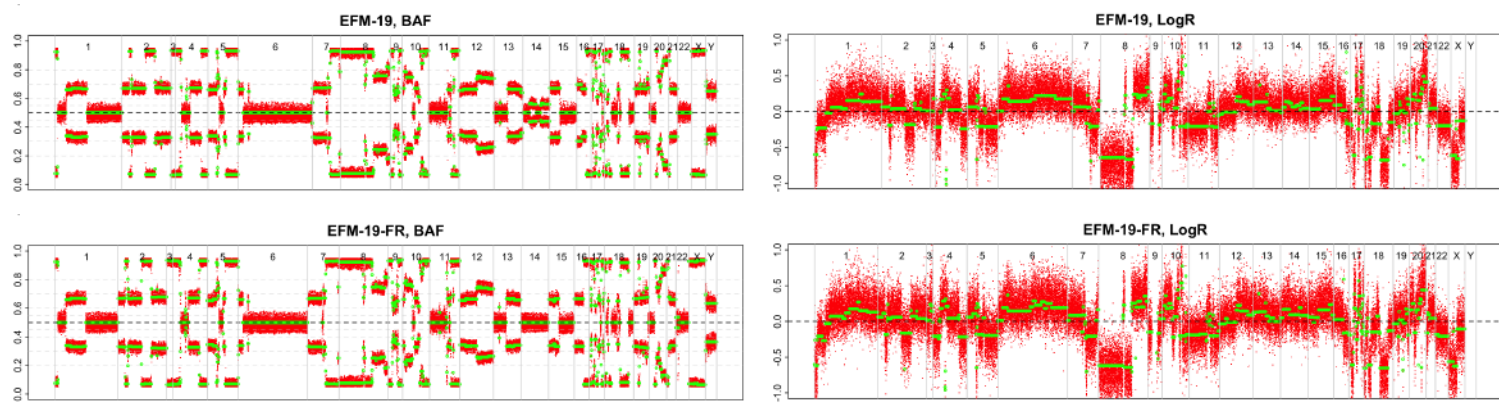

Supplement: Supplementary file 7 — Additional file 7. Figure showing BAF and LogR files for copy number data. B allele frequency (BAF) and LogR genome-wide plots for SNP profiled cell line data presented in Fig. 4a. Chromosomes are ordered along the x-axis from 1 (left) to Y (right). Green lines represent segments derived from ASCAT 2 segmentation of the data. [file 13058_2021_1402_MOESM7_ESM.pdf]

**A**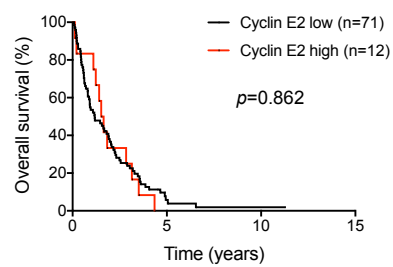**B**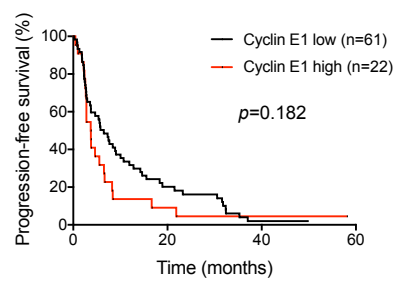**C**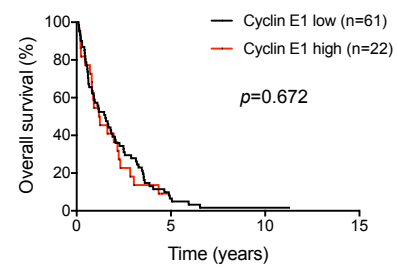

Supplement: Supplementary file 10 — Additional file 10. Figure showing survival analyses of cyclin E1 and E2 in fulvestrant-treated metastatic breast cancer patients. Kaplan-Meier plot for overall survival (OS) in cyclin E2 low versus high expression in ER+ metastatic breast cancer patients treated with fulvestrant in the advanced setting (A) and for progression-free (PFS) (B) and overall (C) survival in cyclin E1 low versus high expression in the same patient material. P-value represents log-rank test for OS and PFS, respectively, between patients with high and low levels of cyclin E1 or E2 expression. [file 13058_2021_1402_MOESM10_ESM.pdf]
