# Supplementary material for: Distinct mechanisms of resistance to fulvestrant treatment dictate level of ER independence and selective response to CDK inhibitors in metastatic breast cancer
Source: Breast Cancer Res. 2021 Feb 18;23:26. doi: 10.1186/s13058-021-01402-1 (PMC7893923; doi:10.1186/s13058-021-01402-1)
Supplement: Supplementary file 8 — Additional file 8. Table showing common processes enriched in fulvestrant-resistant cells identified by DAVID bioinformatics tool. DAVID functional categories and gene ontology (GO) functions as well as KEGG and Biocarta pathways for genes downregulated upon fulvestrant treatment in parental cells and then upregulated again upon development of resistance. The first table show data for CAMA-1, MCF7 and T47D and the second table show data HCC1428. [file 13058_2021_1402_MOESM8_ESM.pdf]

Common processes enriched in fulvestrant-resistant CAMA-1, MCF7 and T47D identified by DAVID bioinformatics tool.

**Enriched gene ontology function for correlated genes**					
**Annotation cluster 1****	**Enrichment score: 22.9**				
**Category**	**Term**	**Count**	**% ***	**p-value**	**Benjamini**
**UP_KEYWORDS**	Cell cycle	47	33.6	1.8E-34	3.0E-32
**UP_KEYWORDS**	Cell division	32	22.9	1.4E-24	7.5E-23
**GOTERM_BP_DIRECT**	cell division	31	22.1	2.5E-23	7.0E-21
**UP_KEYWORDS**	Mitosis	25	17.9	1.6E-20	4.3E-19
**GOTERM_BP_DIRECT**	mitotic nuclear division	21	15.0	3.3E-15	3.7E-13
**Annotation Cluster 2****	**Enrichment Score: 10.69**				
**Category**	**Term**	**Count**	**% ***	**p-value**	**Benjamini**
**GOTERM_BP_DIRECT**	DNA repair	19	13.6	2.1E-13	2.0E-11
**UP_KEYWORDS**	DNA damage	19	13.6	2.9E-11	4.3E-10
**UP_KEYWORDS**	DNA repair	16	11.4	1.4E-9	1.7E-8
**Annotation Cluster 3****	**Enrichment Score: 9.58**				
**Category**	**Term**	**Count**	**% ***	**p-value**	**Benjamini**
**UP_KEYWORDS**	Chromosome	24	17.1	3.3E-15	7.6E-14
**UP_KEYWORDS**	Centromere	14	10.0	9.0E-12	1.5E-10
**GOTERM_BP_DIRECT**	sister chromatid cohesion	13	9.3	1.9E-11	1.6E-9
**GOTERM_CC_DIRECT**	condensed chromosome kinetochore	10	7.1	1.3E-8	3.8E-7
**UP_KEYWORDS**	Kinetochore	10	7.1	2.3E-8	2.6E-7
**GOTERM_CC_DIRECT**	kinetochore	8	5.7	2.0E-6	4.3E-5
**Enriched KEGG pathways for correlated genes*****					
**Category**	**Term**	**Count**	**% ***	**p-value**	**Benjamini**
**KEGG_PATHWAY**	DNA replication	15	10.7	1.3E-21	8.7E-20
**KEGG_PATHWAY**	Cell cycle	18	12.9	1.2E-17	4.3E-16
**KEGG_PATHWAY**	Mismatch repair	6	4.3	6.6E-7	1.5E-5
**KEGG_PATHWAY**	Base excision repair	6	4.3	4.4E-6	7.6E-5
**KEGG_PATHWAY**	Pyrimidine metabolism	8	5.7	1.2E-5	1.7E-4
**KEGG_PATHWAY**	Nucleotide excision repair	6	4.3	2.6E-5	3.0E-4
**KEGG_PATHWAY**	p53 signaling pathway	6	4.3	1.5E-4	1.5E-3
**KEGG_PATHWAY**	Fanconi anemia pathway	5	3.6	6.8E-4	5.9E-3
**KEGG_PATHWAY**	Purine metabolism	7	5.0	2.1E-3	1.6E-2
**KEGG_PATHWAY**	Small cell lung cancer	5	3.6	3.9E-3	2.7E-2
**Enriched BIOCARTA pathways for correlated genes******					
**Category**	**Term**	**Count**	**% ***	**p-value**	**Benjamini**
**BIOCARTA**	CDK Regulation of DNA Replication	9	6.4	7.4E-11	2.8E-9
**BIOCARTA**	Cyclins and Cell Cycle Regulation	5	3.6	6.0E-4	1.1E-2

* Gene list of regulated genes that follow the analysed pattern contains 160 significant genes, out of which 140 genes were recognised by DAVID and used in the % calculation.
** 24 annotation clusters were identified using standard settings, the three most enriched annotation clusters are included.
*** KEGG pathway analysis yielded 19 chart records, entries with Benjamini ≤ 0.05 are included.
**** BIOCARTA analysis yielded seven chart entries, entries with Benjamini ≤ 0.05 are included.

DAVID functional categories and gene ontology (GO) functions as well as KEGG and Biocarta pathways for genes commonly downregulated upon fulvestrant treatment in parental cells and then upregulated again upon development of resistance.

Common processes enriched in fulvestrant-resistant HCC1428 identified by DAVID bioinformatics tool.

**Enriched gene ontology function for correlated genes**					
**Annotation cluster 1****	**Enrichment score: 19.6**				
**Category**	**Term**	**Count**	**% ***	**p-value**	**Benjamini**
**UP_KEYWORDS**	DNA replication	38	7.7	6.6E-37	2.1E-34
**GOTERM_BP_DIRECT**	DNA replication	42	8.6	5.6E-31	8.6E-28
**GOTERM_BP_DIRECT**	DNA replication initiation	17	3.5	5.3E-18	4.1E-15
**GOTERM_BP_DIRECT**	G1/S transition of mitotic cell cycle	25	5.1	2.6E-17	1.3E-14
**Annotation Cluster 2****	**Enrichment Score: 10.73**				
**Category**	**Term**	**Count**	**% ***	**p-value**	**Benjamini**
**UP_KEYWORDS**	DNA damage	36	7.3	1.0E-13	6.6E-12
**UP_KEYWORDS**	DNA repair	30	6.1	1.6E-11	8.5E-10
**GOTERM_BP_DIRECT**	DNA repair	25	5.1	3.9E-9	7.5E-7
**Annotation Cluster 3****	**Enrichment Score: 8.86**				
**Category**	**Term**	**Count**	**% ***	**p-value**	**Benjamini**
**GOTERM_BP_DIRECT**	DNA synthesis involved in DNA repair	14	2.9	8.8E-13	2.3E-10
**GOTERM_BP_DIRECT**	strand displacement	11	2.2	2.7E-10	5.9E-8
**GOTERM_BP_DIRECT**	regulation of signal transduction by p53 class mediator	14	2.9	1.1E-5	1.5E-3
**Enriched KEGG pathways for correlated genes*****					
**Category**	**Term**	**Count**	**% ***	**p-value**	**Benjamini**
**KEGG_PATHWAY**	DNA replication	19	3.9	2.6E-20	5.1E-18
**KEGG_PATHWAY**	Purine metabolism	23	4.7	2.2E-10	2.2E-8
**KEGG_PATHWAY**	Cell cycle	19	3.9	9.3E-10	6.1E-8
**KEGG_PATHWAY**	Fanconi anemia pathway	13	2.6	3.6E-9	1.8E-7
**KEGG_PATHWAY**	Pyrimidine metabolism	16	3.3	1.8E-8	7.2E-7
**KEGG_PATHWAY**	Homologous recombination	8	1.6	4.7E-6	1.5E-4
**KEGG_PATHWAY**	Base excision repair	8	1.6	1.2E-5	3.3E-4
**KEGG_PATHWAY**	Mismatch repair	7	1.4	1.4E-5	3.5E-4
**KEGG_PATHWAY**	Nucleotide excision repair	8	1.6	1.3E-4	2.8E-3
**Enriched BIOCARTA pathways for correlated genes******					
**Category**	**Term**	**Count**	**% ***	**p-value**	**Benjamini**
**BIOCARTA**	CDK Regulation of DNA Replication	9	1.8	5.4E-9	4.8E-7
**BIOCARTA**	Role of BRCA1, BRCA2 and ATR in Cancer Susceptibility	7	1.4	1.5E-5	6.7E-4
**BIOCARTA**	BRCA1-dependent Ub-ligase activity	4	0.8	9.4E-4	2.8E-2

* Gene list of regulated genes that follow the analysed pattern contains 533 significant genes, out of which 491 genes were recognised by DAVID and used in the % calculation.
** 75 annotation clusters were identified using standard settings, the three most enriched annotation clusters are included.
*** KEGG pathway analysis yielded 14 chart records, entries with Benjamini ≤ 0.05 are included.
**** BIOCARTA analysis yielded five chart entries, entries with Benjamini ≤ 0.05 are included.

DAVID functional categories and gene ontology (GO) functions as well as KEGG and Biocarta pathways for genes commonly downregulated upon fulvestrant treatment in parental cells and then upregulated again upon development of resistance.
