# Supplementary material for: Distinct mechanisms of resistance to fulvestrant treatment dictate level of ER independence and selective response to CDK inhibitors in metastatic breast cancer
Source: Breast Cancer Res. 2021 Feb 18;23:26. doi: 10.1186/s13058-021-01402-1 (PMC7893923; doi:10.1186/s13058-021-01402-1)
Supplement: Supplementary file 9 — Additional file 9. Figure showing cell cycle regulation and response to CDK inhibitors. A) Cell cycle profiles for parental (-P) and fulvestrant-resistant (-FR) CAMA-1 and MCF7 cells showing the proportion of cells in G0/G1, S, and G2/M phase, respectively, after treatment with 100 nM fulvestrant for 72 h (+F) or untreated (ctrl). Combined data from two biological replicates. Statistical differences between the proportion of cells in G0/G1-phase were calculated using student’s t-test. * represents p-value ≤0.05, ns represents no statistical differences. B) Western blotting for expression of cell cycle regulating proteins in parental and fulvestrant-resistant T47D and EFM-19 cells after treatment for 24 h with 100 nM fulvestrant (+F) or DMSO control (-F). Representative data from three biological replicates. β-actin was used as loading control. Quantification of band intensities is presented in Additional file 4 L-M. C) Palbociclib IC50 values in parental and fulvestrant-resistant cells. Calculated from graphs presented in (D) and in Fig. 5b. D) Dose-response curves for parental and fulvestrant-resistant T47D and EFM-19 cells in response to 6-days treatment with increasing concentrations of the CDK4/6 inhibitor palbociclib (1 nM to 10 μM). Graphs represent combined data (average ± SEM) from three biological experiments with three technical replicates each. IC50 values are shown in (C). E) CDKi3 IC50 values in parental and fulvestrant-resistant cells. Calculated from graphs presented in (F) and in Fig. 5c. F) Dose-response curves for parental and fulvestrant-resistant T47D and EFM-19 cells in response to 6-days treatment with increasing concentrations of the CDK1/2 inhibitor CDKi3 (100 pM to 50 μM). Graphs represent combined data (average ± SEM) from three biological experiments with three technical replicates each. IC50 values are shown in (E). G) Time in weeks needed for each parental and fulvestrant-resistant cell line to develop resistance to palbociclib, counted [file 13058_2021_1402_MOESM9_ESM.pdf]

A

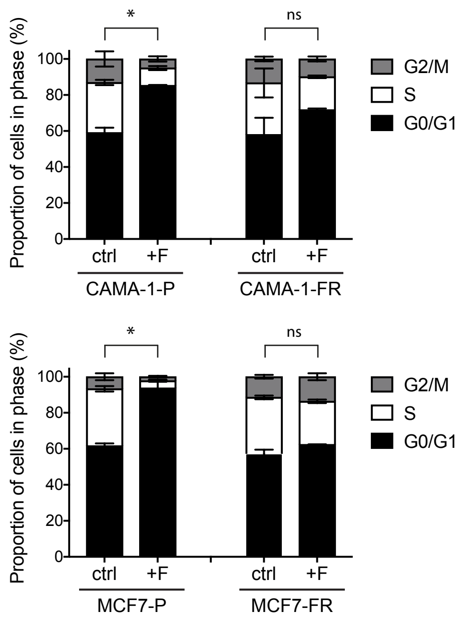

B

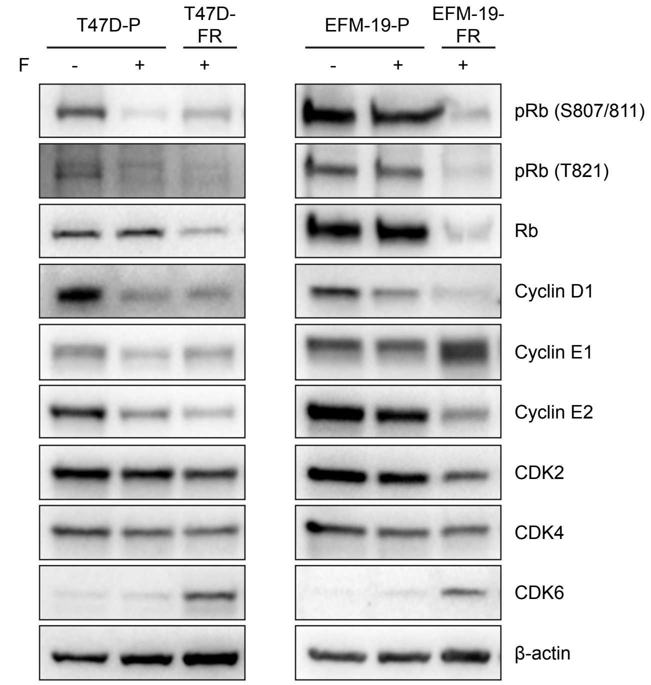

C

IC50 VALUES PALBOCICLIB

	Parental	FR	*p*-value
**CAMA-1**	130 nM	511 nM	<0.0001
**MCF7**	116 nM	134 nM	0.477
**HCC1428**	446 nM	105 nM	<0.0001
**ZR-75-1**	200 nM	50 nM	<0.0001
**T47D**	79 nM	41 nM	0.038
**EFM-19**	62 nM	22 nM	0.0001

D

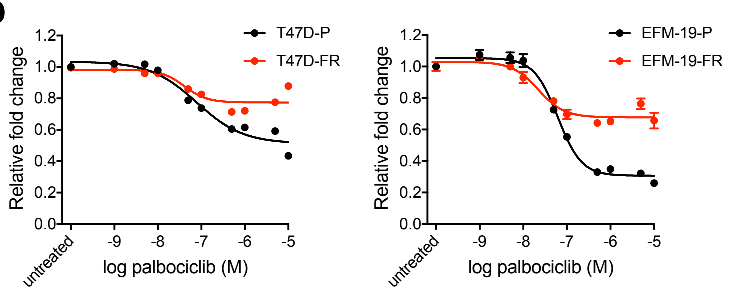

E

IC50 VALUES CDKi3

	Parental	FR	*p*-value
**CAMA-1**	4 nM	15 nM	<0.0001
**MCF7**	26 nM	161 nM	<0.0001
**HCC1428**	156 nM	896 nM	<0.0001
**ZR-75-1**	111 nM	79 nM	0.006
**T47D**	113 nM	763 nM	<0.0001
**EFM-19**	22 nM	225 nM	<0.0001

F

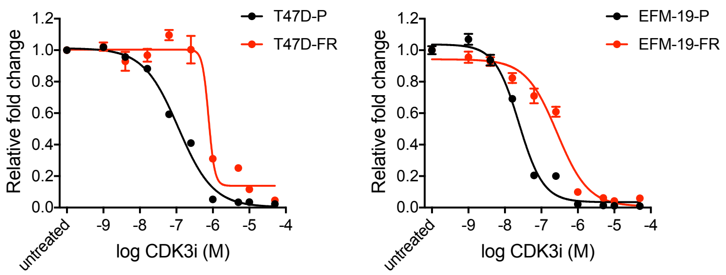

G

TIME TO DEVELOPE PALBOCICLIB RESISTANCE

	Weeks to resistance
**CAMA-1**	20
**CAMA-1-FR**	19
**MCF7**	11.5
**MCF7-FR**	22

H

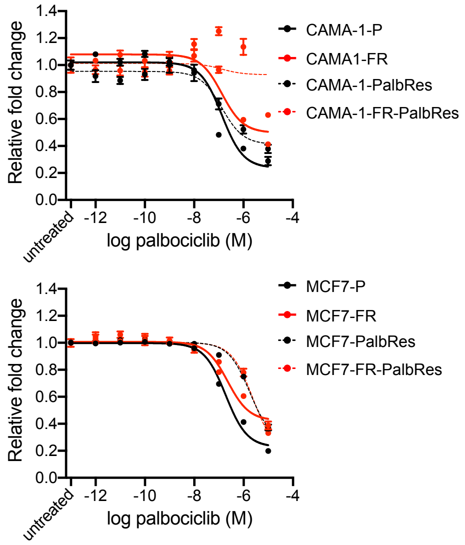

I

IC50 VALUES PALBOCICLIB

	IC50	*p*-value
**CAMA-1-P**	43 nM	
**CAMA-1-FR**	535 nM	<0.0001
**CAMA-1-PalbRes**	161 nM	<0.0001
**CAMA-1-FR-PalbRes**	>10 μM	*cannot be calculated*
**MCF7-P**	187 nM	
**MCF7-FR**	224 nM	0.5459
**MCF7-PalbRes**	1.8 μM	<0.0001
**MCF7-FR-PalbRes**	2.4 μM	<0.0001

J

IC50 VALUES FULVESTRANT

	IC50	*p*-value
**CAMA-1-P**	220 pM	
**CAMA-1-FR**	2.0 μM	<0.0001
**CAMA-1-PalbRes**	911 pM	<0.0001
**CAMA-1-FR-PalbRes**	3.4 μM	<0.0001
**MCF7-P**	690 pM	
**MCF7-FR**	1.6 μM	<0.0001
**MCF7-PalbRes**	593 pM	0.4582
**MCF7-FR-PalbRes**	1.6 μM	0.0001

Additional file 9
